# Three‐Phase‐Heterojunction Cu/Cu_2_O–Sb_2_O_3_ Catalyst Enables Efficient CO_2_ Electroreduction to CO and High‐Performance Aqueous Zn–CO_2_ Battery

**DOI:** 10.1002/advs.202306858

**Published:** 2024-02-27

**Authors:** Junjie Ma, Fang Huang, Aihao Xu, Dong Wei, Xiangyu Chen, Wencan Zhao, Zhengjun Chen, Xucai Yin, Jinliang Zhu, Huibing He, Jing Xu

**Affiliations:** ^1^ School of Chemistry and Chemical Engineering Guangxi Key Laboratory of Petrochemical Resource Processing and Process Intensification Technology, Guangxi Key Laboratory of Electrochemical Energy Materials Guangxi University Nanning 530004 P. R. China; ^2^ School of Resources, Environment, and Materials Collaborative Innovation Center of Sustainable Energy Materials Guangxi Key Laboratory of Processing for Non‐Ferrous Metals and Featured Materials Guangxi University Nanning 530004 P. R. China

**Keywords:** Carbon neutral, CO_2_ electroreduction, DFT calculation, three‐phase heterojunction, Zn–CO_2_ battery

## Abstract

Zn–CO_2_ batteries are excellent candidates for both electrical energy output and CO_2_ utilization, whereas the main challenge is to design electrocatalysts for electrocatalytic CO_2_ reduction reactions with high selectivity and low cost. Herein, the three‐phase heterojunction Cu‐based electrocatalyst (Cu/Cu_2_O‐Sb_2_O_3_‐15) is synthesized and evaluated for highly selective CO_2_ reduction to CO, which shows the highest faradaic efficiency of 96.3% at −1.3 V versus reversible hydrogen electrode, exceeding the previously reported best values for Cu‐based materials. In situ spectroscopy and theoretical analysis indicate that the Sb incorporation into the three‐phase heterojunction Cu/Cu_2_O‐Sb_2_O_3_‐15 nanomaterial promotes the formation of key ^*^COOH intermediates compared with the normal Cu/Cu_2_O composites. Furthermore, the rechargeable aqueous Zn–CO_2_ battery assembled with Cu/Cu_2_O‐Sb_2_O_3_‐15 as the cathode harvests a peak power density of 3.01 mW cm^−2^ as well as outstanding cycling stability of 417 cycles. This research provides fresh perspectives for designing advanced cathodic electrocatalysts for rechargeable Zn–CO_2_ batteries with high‐efficient electricity output together with CO_2_ utilization.

## Graphical abstract

This work proposes an advanced cathodic electrocatalyst of three‐phase heterojunction Cu‐based catalyst (Cu/Cu_2_O‐Sb_2_O_3_‐15) for rechargeable Zn–CO_2_ batteries with high‐efficient electricity output together with CO_2_ utilization.

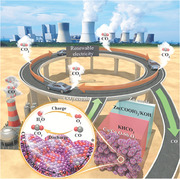

## 1 Introduction

Excessive use of traditional fossil fuels has caused a serious energy crisis and the greenhouse effect of CO_2_, which has led to numerous endeavors to achieve carbon neutrality.^[^ ^1^ ^]^ Electrocatalytic CO_2_ reduction reaction (ECO_2_RR), which utilizes renewable energy to generate electricity, is a potential remedy due to its great energy effectiveness, product diversity, and wide range of applications.^[^ ^2^ ^]^ Nevertheless, the reported ECO_2_RR devices typically require an electrical input to drive the CO_2_ conversion. Therefore, metal–CO_2_ (M‐CO_2_) batteries, as a new technology with both power storage and CO_2_ fixation/utilization functions while outputting electricity and higher value‐added products, began to trigger extensive research by more and more scholars.^[^ ^3^ ^]^ This highly promising energy storage technology could also potentially be applied to Mars exploration.^[^ ^4^ ^]^

However, despite great progress, these metal–CO_2_ batteries (such as Li–CO_2_ and Na–CO_2_ batteries) continue to suffer from poor safety and high cost.^[^ ^5^ ^]^ In contrast, the aqueous Zn–CO_2_ batteries (ZCBs) achieve flexible CO_2_ electrochemistry and energy storage based on a proton‐coupled electron transfer mechanism.^[^ ^6^ ^]^ The aqueous ZCBs can not only catalyze the transformation of CO_2_ into value‐added chemicals such as CO and HCOOH but also mitigate the accumulation of solid products resulting in a durable cycle life.^[^ ^7^ ^]^ Meanwhile, to ensure the rechargeability of aqueous ZCBs, there is an urgent need for a bifunctional catalyst, i.e., CO_2_ reduction during the discharge process and water oxidation to produce O_2_ during the charging process.^[^ ^8^ ^]^ Although the two reactions have been studied separately on multiple catalysts in half cells, little research has been done on bifunctional catalysts for both reactions.^[^ ^9^ ^]^ So far only several multipurpose catalysts have been designed for aqueous ZCBs, including noble metal‐based materials, non‐noble metal‐based materials, and carbon‐based materials.^[^ ^10^ ^]^ Among them, non‐noble metal‐based materials deserve further research due to their cheapness and abundant reserves. As one of the key components of non‐precious metal‐based materials, Cu‐based catalysts have received widespread attention as excellent candidates for ECO_2_RR because of a variety of valuable chemical generation in the electrocatalytic process.^[^ ^11^ ^]^ In the recent study, Xie et al. described an aqueous Pd‐doped La_2_CuO_4_‐based reversible ZCB for CO_2_ to CO conversion featuring a maximal power density of 0.75 mW cm^−2^.^[^ ^12^ ^]^ Meanwhile, a reversible ZCB with a cycle life of up to 40 h was created by Zheng et al. using Cu–N_2_/GN as the cathode.^[^ ^13^ ^]^ However, it is worth noting that these batteries with Cu‐based catalysts as cathodes typically exhibit low power densities as well as poor cycle lifetime, which makes their practical application challenging.^[^ ^14^ ^]^ Therefore, it is urgent to optimize the Cu‐based ECO_2_RR catalysts in ZCBs to generate increased energy density as well as prolonged cycle life.

Herein, the heterojunction Cu‐based electrocatalysts first synthesized by a facile hydrothermal method (Cu/Cu_2_O‐Sb_2_O_3_‐*Y*, where *Y* is the molar concentration ratio of Sb, Cu) were reported and served as the excellent catalysts for efficient electrocatalytic conversion of CO_2_ to CO. As a result, the best three‐phase heterojunction Cu/Cu_2_O‐Sb_2_O_3_‐15 achieved up to 96.3% FE_CO_ at a partial current density of −18.76 mA cm^−2^ at −1.3 V versus reversible hydrogen electrode (vs RHE, all potentials were uncorrected for *iR*). Compared with Cu/Cu_2_O, the Cu/Cu_2_O‐Sb_2_O_3_‐15 had stronger inhibition on by‐products, larger electrochemical surface area (ECSA), lower charge‐transfer resistance (*R*_CT_), smaller Tafel slope, and higher Faraday efficiency value for CO. More importantly, the rechargeable aqueous ZCB with Cu/Cu_2_O‐Sb_2_O_3_‐15 as the cathode exhibited the maximum power density of 3.01 mW cm^−2^ and the charge/discharge stability of more than 150 h, which is superior to that of the recently reported ZCBs (see details in Table S1, Supporting Information). Meanwhile, the prepared battery could be used to produce high additional fuel CO at a charging current density of 3 mA cm^−2^. The density functional theory (DFT) calculations and in situ attenuated total reflection infrared (in situ ATR‐IR) spectroscopy synergistically demonstrated that the three‐phase heterojunction Cu/ Cu_2_O‐Sb_2_O_3_‐15 can effectively reduce the formation energy barrier of the reaction intermediate ^*^COOH, thus enhancing the charging‐discharging efficiency of the rechargeable ZCBs. This study provides a promising strategy for the high‐efficiency utilization of CO_2_ in rechargeable ZCBs with low‐cost Cu‐based cathodic catalysts.

## 2 Results and Discussion

### 2.1 Synthesis and Characterization of Catalysts

**Figure 1:**
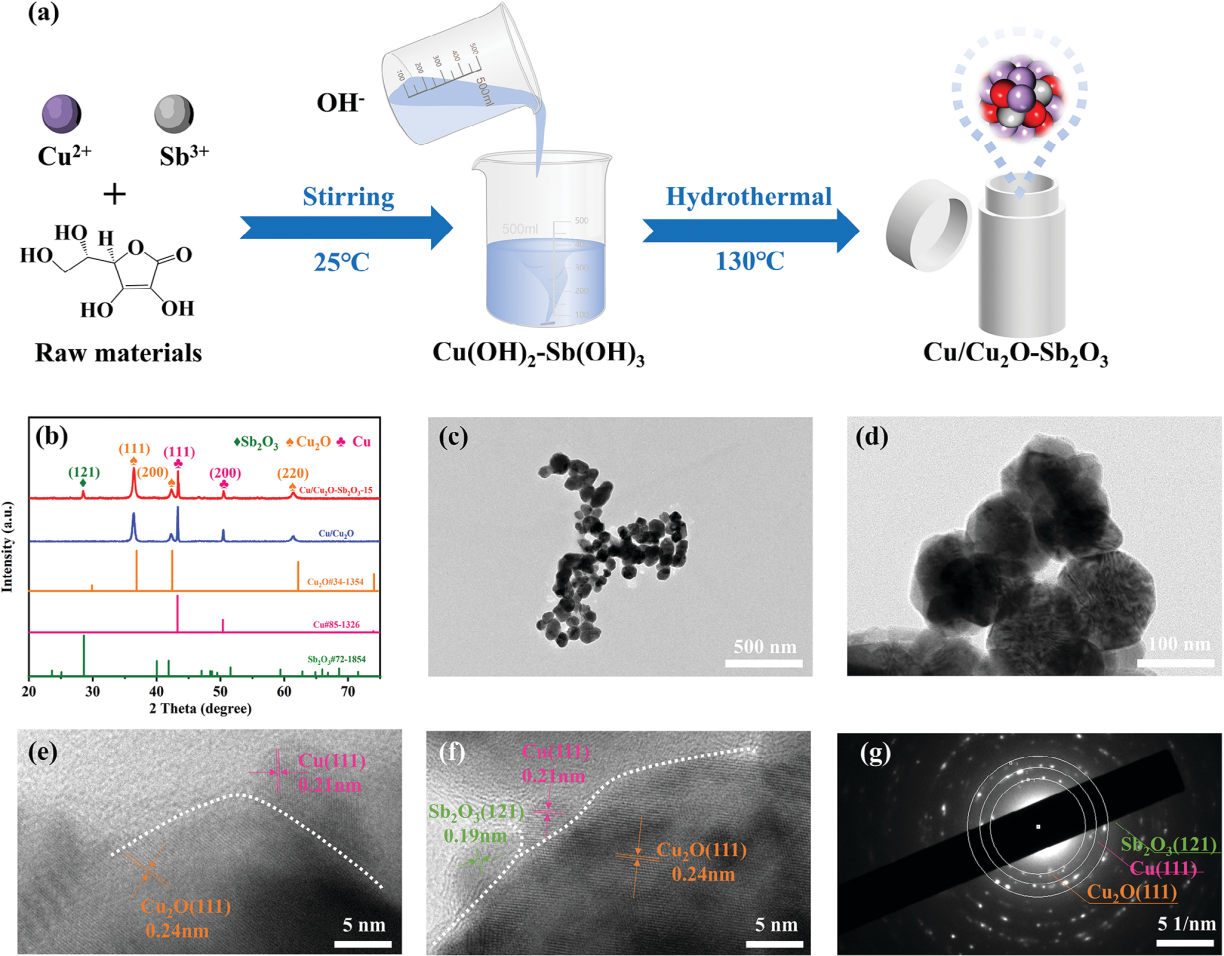
Materials preparation and structure characterizations for the catalysts. a) Schematic diagram illustrating the preparation procedure of Cu/Cu_2_O‐Sb_2_O_3_. b) XRD patterns of Cu/Cu_2_O and Cu/Cu_2_O‐Sb_2_O_3_‐15. c,d) TEM images of Cu/Cu_2_O‐Sb_2_O_3_‐15. HRTEM image of e) Cu/Cu_2_O and f) Cu/Cu_2_O‐Sb_2_O_3_‐15. g) SAED pattern of Cu/Cu_2_O‐Sb_2_O_3_‐15.

As shown in **Figure** **1**a, the Cu/Cu_2_O‐Sb_2_O_3_ catalysts were synthesized by simple hydrothermal reactions. The final Sb/Cu molar proportion in the catalysts was determined by ICP‐OES (Table S2, Supporting Information) and it was obvious that the data was almost in general agreement with the feedstock proportion of Sb/Cu. The XRD plots of Cu/Cu_2_O‐Sb_2_O_3_‐Y with different molar concentration ratios are shown in Figure 1b and Figure S1 (Supporting Information). Figure 1b shows the two main diffraction peaks at 43.32° and 51.15° correspond to the (111) and (200) crystallographic planes of Cu (PDF#85‐1326) respectively, and the three main diffraction peaks at 37.01°, 42.63°, and 62.44° correspond to the (111), (200), and (220) crystallographic planes of Cu_2_O (PDF#34‐1354), respectively. Similarly, another peak located at 28.39^°^ was identified as the (121) plane of Sb_2_O_3_ (PDF#72‐1854), indicating the successful preparation of the three‐phase heterojunction Cu/Cu_2_O‐Sb_2_O_3_‐15 catalyst. Furthermore, the crystal phase amount of Sb_2_O_3_, Cu_2_O, and Cu in the three‐phase heterojunction Cu/Cu_2_O‐Sb_2_O_3_‐15 catalyst was estimated to be 11%, 57%, and 32%, respectively (Table S3, Supporting Information).

The TEM of Cu/Cu_2_O‐Sb_2_O_3_‐15 (Figure 1c,d, and Figure S3a, Supporting Information) presented a very similar loose nanosphere aggregates morphology but with a larger particle size compared to that of Cu/Cu_2_O (Figure S3b, Supporting Information). The lattice spacing of Cu/Cu_2_O was verified by high‐resolution transmission electron microscopy and the fast Fourier transform (FFT) was performed for the regions with a lattice spacing of 0.21 and 0.24 nm in Figure 1e. The results showed that the 0.21 nm lattice spacing corresponds to the Cu (111) crystal plane, while the 0.24 nm lattice spacing corresponds to the Cu_2_O (111) crystal plane, and the two‐phase interface of the Cu/Cu_2_O catalyst can also be seen in Figure 1e. Similarly, the lattice spacings of 0.19, 0.21, and 0.24 nm (Figure 1f) were demonstrated by FFT to correspond to the Sb_2_O_3_ (121), Cu (111), and Cu_2_O (111) crystal planes, respectively, while the three‐phase interface of the Cu/Cu_2_O‐Sb_2_O_3_‐15 catalyst was visible. In addition, the presence of Cu (111), Cu_2_O (111), and Sb_2_O_3_ (121) three‐phase crystallographic surfaces in Cu/Cu_2_O‐Sb_2_O_3_‐15 was further supported by the selected area electron diffraction pattern (Figure 1g). The energy dispersive spectroscopy mapping of Cu/Cu_2_O‐Sb_2_O_3_‐15 confirmed the even distribution of Cu, O, and Sb elements (Figures S4 and S5, Supporting Information). The crystal structure and microscopic morphology results indicate that Sb_2_O_3_ was readily incorporated into the Cu/Cu_2_O, forming a unique three‐phase heterojunction structure that might help improve the corresponding ECO_2_RR performance.

**Figure 2:**
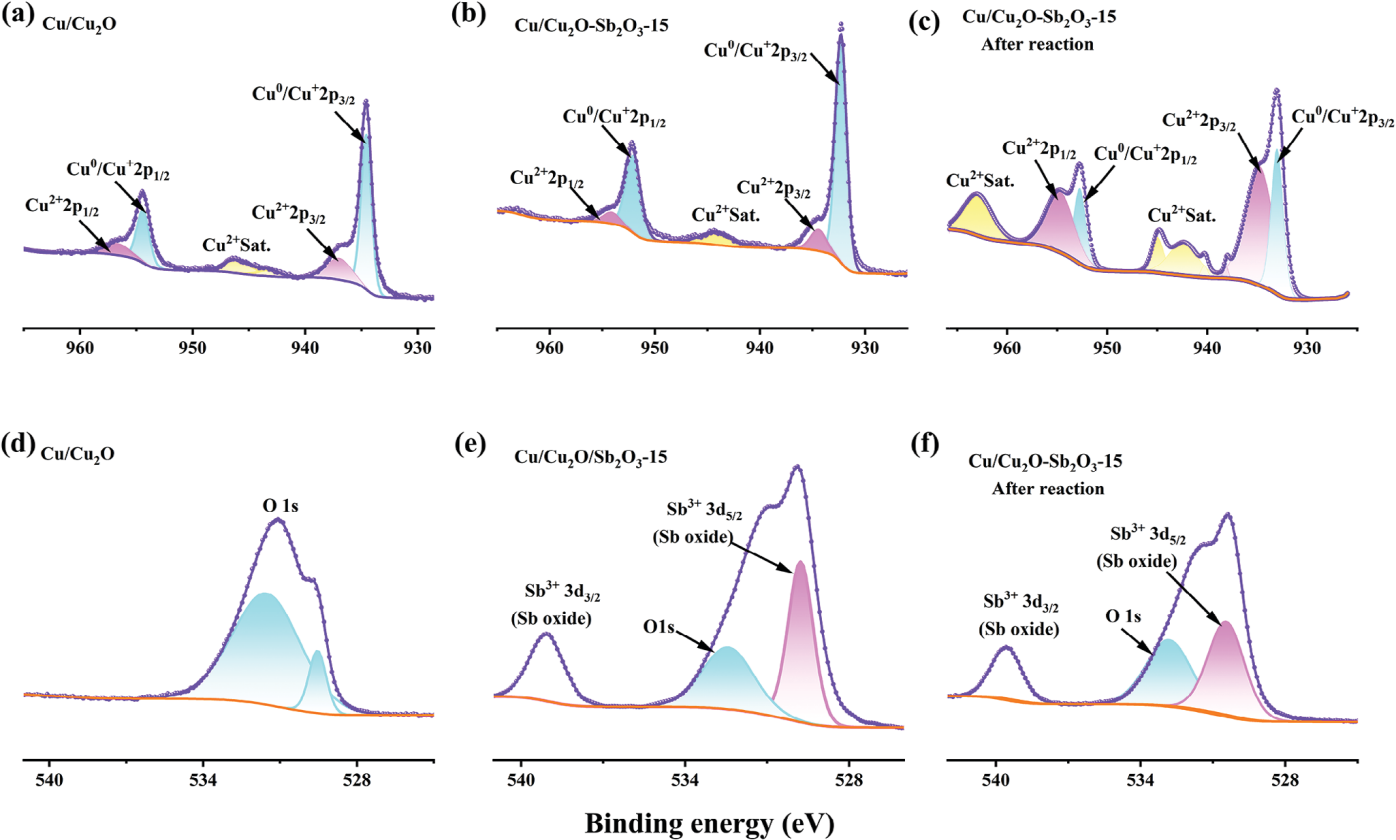
XPS analysis of the prepared catalysts. a) Cu 2p of Cu/Cu_2_O, b) Cu/Cu_2_O‐Sb_2_O_3_‐15, and c) Cu/Cu_2_O‐Sb_2_O_3_‐15 after the reaction. d) O 1s and Sb 3d of Cu/Cu_2_O, e) Cu/Cu_2_O‐Sb_2_O_3_‐15, and f) Cu/Cu_2_O‐Sb_2_O_3_‐15 after the reaction.

The near‐surface chemical states of the samples before and after the electrocatalytic reaction were probed by XPS. Compared with the two‐phase heterojunction Cu/Cu_2_O (Figure S7, Supporting Information), the XPS full spectrum of the three‐phase heterojunction Cu/Cu_2_O‐Sb_2_O_3_‐15 (Figure S6, Supporting Information) showed the presence of Sb, further confirming the successful synthesis of the three‐phase heterojunction Cu/Cu_2_O‐Sb_2_O_3_‐15. Analysis of **Figure** **2**a,b revealed the presence of smaller 2p peaks of Cu^2+^ for both Cu/Cu_2_O‐Sb_2_O_3_‐15 and Cu/Cu_2_O, indicating that the catalyst was oxidized in trace amounts in the natural environment. In Figure 2b, the binding energy at the 2p_3/2_ peak of the three‐phase heterojunction Cu/Cu_2_O‐Sb_2_O_3_‐15 Cu^2+^ was 934.51 eV and the binding energy at 2p_1/2_ was 954.34 eV, respectively, which were shifted toward lower binding energy than Cu_2_O (937.58 eV at 2p_3/2_ and 957.18 eV at 2p_1/2_) (Figure 2a) shifted by 3.07 and 2.84 eV, which indicated that the introduction of Sb_2_O_3_, an electron transfer occurred leading to an increase in the electron density around Cu.^[^ ^15^ ^]^ As Figure 2b showes that the Cu^2+^ characteristic satellite peaks are positioned between 939.58 and 948.73 eV.^[^ ^16^ ^]^ The peaks at binding energies of 932.17 eV and 951.94 eV represented the 2p_3/2_ and 2p_1/2_ peaks of Cu^0^/Cu^+^, respectively. Analogous chemical states of Cu 2p were observed in the two‐phase heterojunction Cu/Cu_2_O (Figure 2a), but the peak intensity of Cu^0^/Cu^+^ was weaker, indicating that antimony oxide played a critical function in stabilizing the activated center (Cu^0^/Cu^+^). Besides, we also conducted XPS detection for the optimal catalyst after the reaction to determine the active center in the catalyst (Figure 2c). Obviously, the peak intensity of Cu^0^/Cu^+^ decreases significantly after the reaction, while Cu^2+^ peak intensity increases significantly, which further indicates that the catalytic activity center was mainly Cu^0^/Cu^+^. Figure 2e showed the obvious Sb 3d peaks in Cu/Cu_2_O‐Sb_2_O_3_‐15 with a binding energy of 538.9 eV for Sb 3d_3/2_ and 529.7 eV for Sb 3d_5/2_. The peaks here represented the 3d peaks of Sb^3+^, thus the presence of trivalent antimony, i.e., Sb_2_O_3_, in the Cu/Cu_2_O‐Sb_2_O_3_‐15 material, instead only the O1s peak appears in Cu/Cu_2_O (Figure 2d). This conclusion complemented the result that TEM and XRD could not determine the valence state of antimony in Cu/Cu_2_O‐Sb_2_O_3_‐15. And the two fitted peaks of the O 1s peak in Cu/Cu_2_O (Figure 2d) represented the lattice oxygen peak and the surface adsorbed oxygen peak, respectively. Furthermore, the peak intensity of Sb─O showed a negligible decrease after the reaction (Figure 2e,f), which implied that Sb_2_O_3_ was very stable as a cocatalyst in the catalytic process.

To further investigate the effect of the introduction of Sb_2_O_3_ on the catalytic activity center of Cu/Cu_2_O, we performed Raman tests on Cu/Cu_2_O‐Sb_2_O_3_‐15 and Cu/Cu_2_O (Figure S8, Supporting Information). Both Cu/Cu_2_O‐Sb_2_O_3_‐15 and Cu/Cu_2_O exhibited two characteristic peaks of Cu_2_O which located at 461 cm^−1^ and 623 cm^−1^.^[^ ^17^ ^]^ Cu/Cu_2_O‐Sb_2_O_3_‐15 showed an additional peak of Sb_2_O_3_ at 324.5 cm^−1^ compared to Cu/Cu_2_O, which represented the stretching vibration of the Sb─O bond, suggesting the presence of Sb^3+^ in Cu/Cu_2_O‐Sb_2_O_3_‐15.^[^ ^18^ ^]^ The Raman test was also consistent with the results of the above XPS analysis. Moreover, the intensity of the peak at 461 cm^−1^ was significantly enhanced after the introduction of Sb_2_O_3_, which further confirmed that the introduction of Sb^3+^ was conducive to the stabilization of the catalytic activity center of Cu^+^.

### 2.2 Evaluation of ECO_2_RR Performance

**Figure 3:**
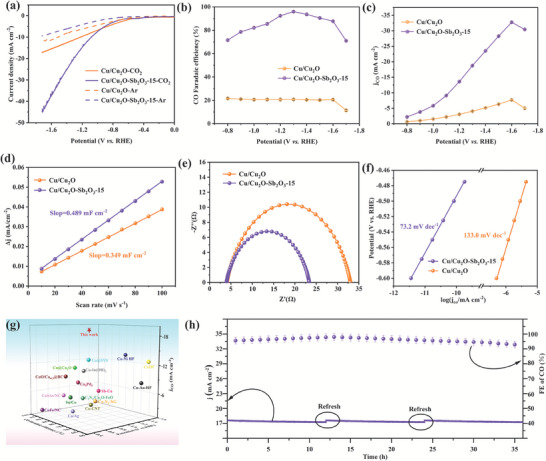
Electrochemical performance evaluation of the catalysts. a) LSV curves were measured in 0.1 m KHCO_3_ solution saturated with Ar or CO_2_ (scanning rate of 5 mV s^−1^), b) FE_CO_, c) subcurrent density CO, d) double‐layer capacitance, e) Nyquist plots, and f) Tafel plots of Cu/Cu_2_O and Cu/Cu_2_O‐Sb_2_O_3_‐15 catalysts. g) comparison of ECO_2_RR activity for Cu/Cu_2_O‐Sb_2_O_3_‐15 with the latest reported Cu‐based catalysts. h) Durability test of Cu/Cu_2_O‐Sb_2_O_3_‐15 catalyst at −1.3 V versus RHE.

The ECO_2_RR activity and performance evaluation of Cu/Cu_2_O–Sb_2_O_3_ catalysts were performed using conventional H‐cells separated by Nafion117 membranes. Among all the tested samples, the three‐phase heterojunction Cu/Cu_2_O‐Sb_2_O_3_‐15 electrode exhibited the best catalytic performance in terms of the Faraday efficiency of CO (FE_CO_) (Figure S11, Supporting Information) and CO partial current density (*j*_CO_) (Figure S12, Supporting Information). In addition, the Cu/Cu_2_O‐Sb_2_O_3_‐15 catalyst showed higher CO_2_RR turnover frequency (TOF) values than the other catalysts with heterogeneous ratios at all potentials (Figure S13, Supporting Information). Therefore, the subsequent electrochemical performance studies were mainly focused on the Cu/Cu_2_O‐Sb_2_O_3_‐15 electrode and Cu/Cu_2_O electrode. Figure 2a shows the LSV curves of Cu/Cu_2_O‐Sb_2_O_3_‐15 and Cu/Cu_2_O under saturated Ar or CO_2_ conditions. It is well known that the measured polarization current density in an Ar‐saturated electrolyte (*j*_Ar_) can be attributed exclusively to hydrogen evolution reaction (HER), whereas that tested in a CO_2_‐saturated electrolyte (*j*_CO2_) can be ascribed to ECO_2_RR or competing reactions HER. Both Cu/Cu_2_O‐Sb_2_O_3_‐15 and Cu/Cu_2_O showed higher *j*_CO2_ than *j*_Ar_, indicating that the ECO_2_RR occurs readily. Meanwhile, the current density of the Cu/Cu_2_O‐Sb_2_O_3_‐15 electrode under the Ar atmosphere was less than that of Cu/Cu_2_O under the same potential, indicating that the HER side reaction was suppressed after the introduction of the Sb_2_O_3_. In contrast, all the current densities of the two‐phase heterojunction Cu/Cu_2_O under the CO_2_ atmosphere were much lower than those of the three‐phase heterojunction Cu/Cu_2_O‐Sb_2_O_3_‐15 at the same potential. Especially, when applied at −1.7 V versus RHE (**Figure** **3**a), the total current density of the Cu/Cu_2_O‐Sb_2_O_3_‐15 electrode potential was as high as 45.45 mA cm^−2^, more than twice that in the Cu/Cu_2_O electrode (17.52 mA cm^−2^). In short, these results strongly demonstrated the remarkable ECO_2_RR performance of the three‐phase heterojunction Cu/Cu_2_O‐Sb_2_O_3_‐15 catalyst as well as the inhibition of the side HER.

To evaluate the product distribution of CO_2_ electroreduction, the gas products at different potentials were determined by gas chromatography. The *I*–*T* tests were run for 1800 s at each potential. As shown in Figure S9 (Supporting Information), the smooth *I*–*T* curves highly prove that Cu/Cu_2_O‐Sb_2_O_3_‐15 can work stably at all potential states. CO and H_2_ were the only two gaseous products of ECO_2_RR detected by gas chromatography. The liquid phase product was also analyzed by ^1^H NMR spectroscopy, showing no peak of the liquid phase product was detected even when local amplification was performed at the position where the liquid phase product was likely to appear (Figure S10, Supporting Information). Furthermore, it can be seen from Figure 3b and Figure S14 (Supporting Information) that the total FE produced by CO and H_2_ was almost 100% in all potential ranges, thus the FE for liquid products was negligible. The Faraday efficiencies of CO (FE_CO_) and H_2_ (FE_H2_) were calculated as shown in Figure 3b and Figure S14 (Supporting Information), respectively, and the partial current densities of CO (*j*_CO_) and H_2_ (*j*_H2_) at specific potentials were also determined in Figure 3c and Figure S15 (Supporting Information) to further evaluate the ECO_2_RR capability. Compared to the two‐phase heterojunction Cu/Cu_2_O electrode, the three‐phase heterojunction Cu/Cu_2_O‐Sb_2_O_3_‐15 electrode exhibited higher FE_CO_ and *j*_CO_, and lower FE_H2_ and *j*_H2_ at all applied potentials. Among them, the FE_CO_ reached a maximum FE of 96.3% at −1.3 V versus RHE with a partial current density of 18.76 mA cm^−2^. Excitingly, even over a relatively wide potential window (from −1.0 to −1.6 V vs RHE), the FE_CO_ remained relatively over 82.1%. Moreover, the *j*_CO_ increased gradually with enlarged potential, reaching a maximum of −32.76 mA cm^−2^ at −1.6 V versus RHE. Furthermore, the *C*_dl_ was calculated from the CV curves at various scan rates and then further analyzed ECSA to further reveal the origin of the enhanced electrocatalytic activity of the three‐phase heterojunction Cu/Cu_2_O‐Sb_2_O_3_‐15 catalyst (Figure S16, Supporting Information). The analysis yielded a larger ECSA for Cu/Cu_2_O‐Sb_2_O_3_‐15 (16.86 cm^2^) than that for Cu/Cu_2_O (12.03 cm^2^) (Figure 3d), implying the higher density of active sites to accelerate the ECO_2_RR performance in the three‐phase heterojunction Cu/Cu_2_O‐Sb_2_O_3_‐15. Further to show the effect of ECSA on the total ECO_2_RR process more intuitively, we performed ECSA normalization of *j*_CO_, and it could be seen in Figure S17 (Supporting Information) that the ECSA‐normalized *j*_CO_ of Cu/Cu_2_O‐Sb_2_O_3_‐15 was much higher than that of Cu/Cu_2_O in the whole range of tested potentials. The Nyquist plots (Figure 3e) with the equivalent circuit in Figure S18 (Supporting Information) disclosed that Cu/Cu_2_O‐Sb_2_O_3_‐15 was characterized by a lower interfacial charge‐transfer resistance (*R*_ct_) than Cu/Cu_2_O, evidencing a faster electron transfer from the electrodes to CO_2_ during the ECO_2_RR process, which should contribute to the higher current density of three‐phase heterojunction Cu/Cu_2_O‐Sb_2_O_3_‐15 than the two‐phase heterojunction Cu/Cu_2_O (Figure 3a,c). The results by Tafel slope showed that the Cu/Cu_2_O‐Sb_2_O_3_‐15 was significantly lower than the Cu/Cu_2_O, which indicates a faster charge transfer kinetic reaction rate (Figure 3f). To further demonstrate the excellent ECO_2_RR performance of the three‐phase heterojunction Cu/Cu_2_O‐Sb_2_O_3_‐15 catalyst, we summarized the ECO_2_RR based on Cu‐based catalysts (Figure 3g), where the three‐phase heterojunction Cu/Cu_2_O‐Sb_2_O_3_‐15 catalyst in this study exhibited the state‐of‐the‐art CO selectivity with higher *j*_CO_ and FE_CO_ over a wide potential window, far exceeding the recently reported Cu‐based catalysts (Table S4, Supporting Information).^[^ ^19^ ^]^

The objective evaluation for catalysts should not only include testing the efficiency but also monitoring the stability. Therefore, we investigated the stability and durability of the optimal Cu/Cu_2_O‐Sb_2_O_3_‐15 catalyst by potentiometric measurement of CO_2_ at −1.3 V versus RHE (Figure 3h). The |*j*_CO_|and FE_CO_ hardly decayed during 36 h of ECO_2_RR (refresh electrolyte every 12 h), indicating that the Cu/Cu_2_O‐Sb_2_O_3_‐15 catalyst has a very promising practical application in long‐term electrocatalysis. Furthermore, the real‐time ex‐situ XRD patterns of the three‐phase heterojunction Cu/Cu_2_O‐Sb_2_O_3_‐15 before and after 36 h of electrolysis at −1.3 V versus RHE are shown in Figure S2 (Supporting Information). It was obvious that the main catalytic active centers still existed in the catalyst after such long‐time electrolysis, which guaranteed the structural stability of the three‐phase heterojunction Cu/Cu_2_O‐Sb_2_O_3_‐15 catalyst.

To further evaluate whether the Cu/Cu_2_O‐Sb_2_O_3_‐15 catalyst could meet the industrial current density criterion (>100 mA cm^−2^), a flow cell equipped with a gas diffusion electrode device was used to probe the ECO_2_RR performance of the Cu/Cu_2_O‐Sb_2_O_3_‐15 catalyst at high current densities. As expected, the polarization current density of Cu/Cu_2_O‐Sb_2_O_3_‐15 observed in the flow cell was much higher than that of the H‐cell (Figure S19, Supporting Information), which not only indicated the superiority of the flow cell but also confirmed that the Cu/Cu_2_O‐Sb_2_O_3_‐15 catalyst can reach industrial catalytic level. Importantly, the FE_CO_ of Cu/Cu_2_O‐Sb_2_O_3_‐15 can still exceed 90% at industrial current densities, with *j*_CO_ showing more than a threefold increase (131.68 vs 35.34 mA cm^−2^ at −1.6 V vs RHE). The electrochemical performance of dual‐phase heterojunction Cu/Cu_2_O in the flow cell was also evaluated with improved overall performance, while it was still far inferior to the three‐phase heterojunction Cu/Cu_2_O‐Sb_2_O_3_‐15 (Figure S20, Supporting Information). And the stability test of the three‐phase heterojunction Cu/Cu_2_O‐Sb_2_O_3_‐15 is up to 16 h at industrial densities close to 150 mA cm^−2^ (Figure S21, Supporting Information). To sum up, the three‐phase heterojunction Cu/Cu_2_O‐Sb_2_O_3_‐15 catalyst has competitive ECO_2_RR performance and great prospects in practical applications.

### 2.3 The ZCB Performance

**Figure 4:**
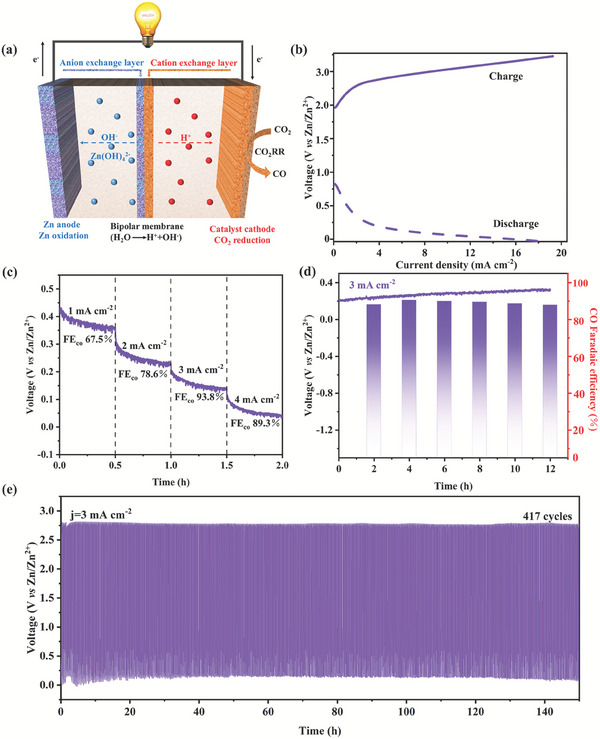
Rechargeable aqueous ZCB performance with Cu/Cu_2_O‐Sb_2_O_3_‐15 as the cathode. a) Schematic diagram of a ZCB with Cu/Cu_2_O‐Sb_2_O_3_‐15 as the cathode. b) Polarization curves for charges and discharges. c) Discharge curves and the related FE_CO_ at various constant current densities. d) FE_CO_ and discharge voltage of Cu/Cu_2_O‐Sb_2_O_3_‐15 were recorded for stability test at 3 mA cm^−2^. e) ZCB charge/discharge cycle curves at a constant current density of 3.0 mA cm^−2^.

As well‐known, flexible, affordable, and beneficial energy conversion and storage are all possible with rechargeable ZCBs. Hence, to broaden the practical use of the Cu/Cu_2_O‐Sb_2_O_3_‐15 electrocatalyst's effective CO_2_‐to‐CO performance, a rechargeable aqueous ZCB was built. The Cu/Cu_2_O‐Sb_2_O_3_‐15 electrocatalyst‐coated electrode was worked in a cathodic electrolyte (1 m KHCO_3_), and a bipolar membrane was employed to separate the two different electrolytes to prevent cross‐contamination. This procedure is shown in **Figure** **4**a. Meanwhile, an anodic electrolyte (6 m KOH with 0.02 m Zn (CH_3_COO)_2_) was added to the polished Zn plate.^[^ ^20^ ^]^ An electrochemical workstation was used to test the ZCB once more, and there, 0.731 V of open‐circuit voltage was found. This value was almost identical to the value of 0.734 V measured by a multimeter (Figure S22, Supporting Information). It could be seen that the calculated theoretical potential was 0.757 V, which was slightly larger than the actual open circuit potential of 0.731 V, which was probably due to the battery resistance causing the potential to drop. The feasibility of the practical application of this ZCB was reflected by its charge/discharge voltage curves (Figure 4b). It was evident that the ZCB had a large charge/discharge voltage gap of 2.25 V at a current density of 2 mA cm^−2^, which only increased slowly to 2.36 V when the current density increased to 4 mA cm^−2^, demonstrating the considerable rechargeability of the ZCB. Notably, the ZCB peak power density can reach up to 3.01 mW cm^−2^ (Figure S23, Supporting Information). Furthermore, the ZCB with Cu/Cu_2_O‐Sb_2_O_3_‐15 cathode showed stable voltage plateaus of ≈0.35, 0.24, 0.16, and 0.05 V with FE_CO_ values of 67.5%, 78.6%, 93.8%, and 89.3% for 2 h at discharge current densities of 1, 2, 3, and 4 mA cm^−2^, respectively, which undoubtedly proved the excellent discharge performance of the assembled ZCB (Figure 4c). Importantly, as shown in Figure 4d, this ZCB could maintain a stable discharge voltage of about 0.24 V for 12 h at a current density of 3 mA cm^−2^, as well as kept the FE_CO_ above 90%, indicating its promising prospect in the energy storage together with the production of high value‐added fuels.

Further, the constant current cycling stability test was explored (Figure 4e). The assembled ZCB showed a charging potential of 2.75 V as well as a discharging potential of 0.05 V when cycled at a constant current of 3 mA cm^−2^, with a cycling endurance of more than 417 cycles (up to 150 h of cycle time), which showed excellent operation stability in the battery system. In addition, the practical application value of the ZCB with Cu/Cu_2_O‐Sb_2_O_3_‐15 cathode was evaluated, which could provide a stable power supply for an electronic clock (Figure S24, Supporting Information). In light of this, it is anticipated that a rechargeable ZCB with a three‐phase heterojunction Cu/Cu_2_O‐Sb_2_O_3_‐15 as the cathode will have a bright future in the fields of carbon neutrality and novel energy storage.

### 2.4 DFT Calculations and In Situ ATR‐IR Analysis

**Figure 5:**
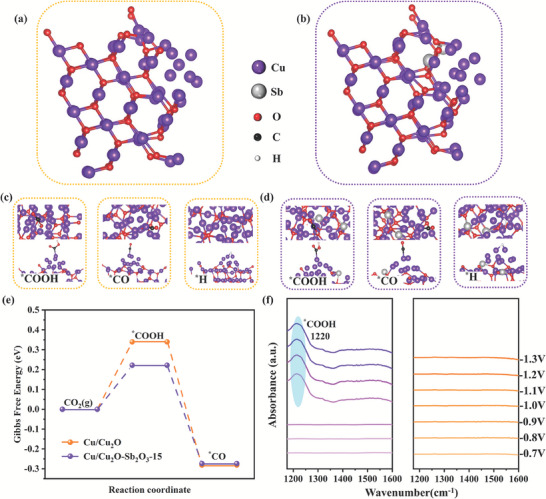
Theoretical calculations and in situ ATR‐IR analysis. Catalysis model of a) Cu/Cu_2_O and b) Cu/Cu_2_O‐Sb_2_O_3_‐15. Front view and top view of adsorption intermediate of ^*^COOH, ^*^CO, and ^*^H on c) Cu/ Cu_2_O and d) Cu/Cu_2_O‐Sb_2_O_3_‐15. e) Free‐energy energy‐barrier diagrams of ECO_2_RR from CO_2_ to ^*^CO intermediates and f) In situ ATR‐IR spectra of Cu/Cu_2_O‐Sb_2_O_3_‐15 and Cu/ Cu_2_O samples were captured with the potential ramped down from −0.7 to −1.3 V versus RHE.



To deeply comprehend the reasons for the enhanced ECO_2_RR performance of Cu/Cu_2_O‐Sb_2_O_3_‐15 catalyst, DFT calculations were performed. As mentioned before, the heterojunction structure was more active for CO_2_RR. The models of the two‐phase heterojunction Cu/Cu_2_O and three‐phase heterojunction Cu/Cu_2_O‐Sb_2_O_3_‐15 were constructed (**Figure** **5**a,b). Different heterostructures for the three‐phase heterojunction Cu/Cu_2_O‐Sb_2_O_3_‐15 were carefully explored and subsequently refined to yield the most stable structure with the lowest formation energy (Figure S25 and Table S5, Supporting Information). In general, the reduction of CO_2_ to CO underwater phase conditions mainly happened in the following three basic processes:^[^ ^21^ ^]^

(1)

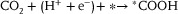



(2)

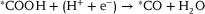



(3)

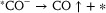




Which * represents the active site which can absorb the species on the catalyst surface. From the above reaction paths, it can be inferred that the free energy barriers of the two intermediate states ^*^COOH and ^*^CO were the essential parameters that determine the effectiveness of ECO_2_RR. The optimal adsorption models of intermediates on Cu/Cu_2_O and Cu/Cu_2_O‐Sb_2_O_3_‐15 under the reaction pathway of CO_2_→^*^COOH→^*^CO were in displayed Figure 5c,d, respectively. The free energies of the Cu/Cu_2_O (111) and Cu/Cu_2_O (111)‐Sb_2_O_3_‐15 adsorption intermediates (Figure 5e,f) were estimated from the calculated hydrogen electrode (CHE) model. The primary stage (^*^+ (H^+^ + e^−^) +CO_2_→^*^COOH) was regarded as the RDS for the two surface rates, due to the higher free energy compared to the other steps. The free energy barrier (Δ*G*) at Cu/Cu_2_O (111) for this step was 0.34 eV, indicating that the initial kinetics of the two‐phase heterojunction Cu/Cu_2_O (111) was slow, and thus requiring a higher starting potential to catalyze the reaction. In contrast, the free energy barrier (Δ*G*) of Cu/Cu_2_O (111)‐Sb_2_O_3_‐15 sharply decreased to 0.22 eV in this basic step, indicating a faster initial kinetics on the Cu/Cu_2_O‐Sb_2_O_3_‐15 catalyst, which greatly improved the ECO_2_RR selectivity and catalytic efficiency. Conversely, for the competitive HER, the ^*^H free energy barrier (Δ*G*) was 0.42 eV on Cu/Cu_2_O (Figure S27, Supporting Information), while the formation of ^*^H on Cu/Cu_2_O‐Sb_2_O_3_‐15 required a greater barrier in free energy (Δ*G*), i.e. −0.65 eV, indicating that the Cu/Cu_2_O‐Sb_2_O_3_‐15 catalyst had a better inhibition effect on the side HER after introducing antimony oxide. Furthermore, we also performed the contact angle measurements (Figure S28, Supporting Information). The contact angle of 0.1 m KHCO_3_ on the three‐phase heterojunction Cu/Cu_2_O‐Sb_2_O_3_‐15 (98°) was larger than that of the two‐phase heterojunction Cu/Cu_2_O (77°), showing its hydrophobic nature in inhibiting the hydrogen precipitation side‐reaction, which was complementary to the theoretical calculations. Meanwhile, we also demonstrated the charge change for the formation of ^*^COOH intermediates on the catalyst surface by tracking the Bader charges (Figure S26, Supporting Information), further confirming that the construction of the three‐phase heterojunction optimized the surface charge of the catalyst and thus significantly lowered the energy barrier for the formation of the intermediates. The above results indicated that the three‐phase heterojunction Cu/Cu_2_O‐Sb_2_O_3_‐15 could improve ECO_2_RR to CO performance and inhibited HER, which wa consistent with the previous experimental results. Furthermore, CO TPD measurement was carried out to prove the strong desorption ability of CO within the optimal catalyst. As shown in Figure S29 (Supporting Information), the CO signal detected by Cu/Cu_2_O‐Sb_2_O_3_‐15 was much higher than that of Cu/Cu_2_O, indicating that the CO generated during the catalytic process was more easily desorbed on the surface of the Cu/Cu_2_O‐Sb_2_O_3_‐15 catalyst, which was responsible for the enhanced performance of ECO_2_RR for CO.

To gain further insight into the ECO_2_RR mechanism of the three‐phase heterojunction Cu/Cu_2_O‐Sb_2_O_3_‐15, the in situ ATR‐IR technique was applied to assimilate the evolution of the reaction intermediates in CO_2_‐saturated 0.1 m KHCO_3_ from −0.7 to −1.3 V versus RHE (the in situ ATR‐IR test equipment was shown in Figure S30, Supporting Information). The two‐phase heterojunction Cu/Cu_2_O was chosen as a comparison sample. A major peak was discernible in the sequential IR spectra between 1200 and 1600 cm^−1^. The band at 1220 cm^−1^ was attributed to the ^*^COOH intermediate.^[^ ^22^ ^]^ The intermediate ^*^COOH peak of the three‐phase heterojunction Cu/Cu_2_O‐Sb_2_O_3_‐15 was revealed at −1.0 V versus RHE, whereas the comparison sample had almost no intermediate ^*^COOH peak indicating that the ^*^COOH formation energy of Cu/Cu_2_O‐Sb_2_O_3_‐15 was significantly lower than that of Cu/Cu_2_O (Figure 5f). The afore mentioned in situ ATR‐IR results clearly showed how Cu/Cu_2_O‐Sb_2_O_3_‐15 had a significant advantage of easily producing key ^*^COOH intermediates during the CO_2_ to CO transition, which was well in agreement with the above theoretical calculations.

## 3 Conclusions

In summary, we have developed the three‐phase heterojunction Cu/Cu_2_O‐Sb_2_O_3_‐15 as an effective Cu‐based electrocatalyst with excellent performance in terms of both the high selectivity and stabilization for ECO_2_RR to CO generation and the successful application in rechargeable ZCB as the cathode. With a maximum FE_CO_ of 96.3% vs. RHE and stable operation for 36 h, the three‐phase heterojunction Cu/Cu_2_O‐Sb_2_O_3_‐15 displays outstanding selectivity and endurance. In addition, the Cu/Cu_2_O‐Sb_2_O_3_‐15 as the cathode material fosters the assembled ZCB with a competitive peak power density of 3.01 mW cm^−2^ and stable cycling up to 417 times with excellent ECO_2_RR performance while discharging. The DFT calculations and in situ ATR‐IR characterization jointly reveal the main reason for the superior catalytic performance is ascribed to the facile formation of the key intermediate of ^*^COOH. This research opens fresh possibilities for the fixation and usage of CO_2_ through rechargeable aqueous Zn–CO_2_ batteries, providing new insights into constructing highly efficient electrocatalysts for selective CO_2_ reduction.

## 4 Experimental Section

### Synthesis of the Catalysts

In a typical procedure, 2.416 g Cu (NO_3_)_2_·3H_2_O and 0.342 g SbCl_3_ were dissolved in 50 mL ethanol. Then, an aqueous KOH solution was added dropwise until the Cu hydroxide and antimony hydroxide were completely precipitated. The thoroughly washed precipitate was mixed with 50 mL deionized water and 0.881 g C_6_H_8_O_6_ and then transferred to a 50 mL Teflon polytetrafluoroethylene reactor. The hydrothermal reaction was carried out in a dryer at 130 °C for 12 h. When cooled to 25 °C, the product was rinsed several times with anhydrous ethanol and deionized water to remove unreacted reactants and then dried under vacuum at 60 °C for 12 h. A series of samples of three‐phase heterojunction Cu/Cu_2_O‐Sb_2_O_3_‐*Y* catalysts (*Y* = 5, 10, 15, 20, 25, and 30) were prepared by the same procedure above. For comparison, we also prepared two‐phase heterojunction Cu/Cu_2_O and single‐phase Sb_2_O_3_ to determine the effect of different heterojunction surfaces on CO selectivity.

### Characterization

The element's content of the catalyst was determined by inductively coupled plasma optical emission spectroscopy (ICP‐OES). The crystallographic phase of the catalyst was identified using the X‐ray diffractometer (XRD) with a scan rate of 5° min^−1^ and a step size of 0.01° in the 2*θ* range of 20° to 80°. The near‐surface chemistry of the catalysts was conducted by X‐ray photoelectron spectroscopy (XPS) with a monochromatic Al Kα X‐ray source (1486.6 eV) operating at 100 W. The morphology of the catalysts was examined by field emission scanning electron microscopy (SEM) and transmission electron microscopy (TEM). The programmed temperature rise desorption of CO (CO‐TPD) was performed on a fully automated temperature‐programmed chemisorption instrument. Finally, in situ ATR‐IR spectroscopy was measured in the customized battery (Figure S30, Supporting Information).

### Electrochemical Measurements

The potential was controlled by an electrochemical workstation (CHI 760E). A three‐electrode system was used for the electrolytic cell with a reference electrode (Ag/AgCl), a counter electrode (Pt), and a working electrode (carbon paper loaded with catalyst). The H electrolytic cell consisted of a positive and a negative chamber separated by a proton exchange membrane (Nafion117 proton exchange membrane), and the positive and anode chambers were each filled with 30 mL of electrolyte solution. The flow cell consisted of four rectangular fittings made of peek material, with an effective area of 1 cm^2^ for the catalytic reaction. The reaction was carried out by contacting CO_2_ with the catalyst and electrolyte through a gas diffusion electrode, which was stored in a 100 mL container with the electrolyte flow generated by a peristaltic pump at a rate of 30 mL min^−1^. The Ag/AgCl reference electrode (in potassium chloride solution, 3.5 m) was applied to all potential measurements with *E* (vs RHE) = E (vs Ag/AgCl) + 0.2046 + 0.0591 × pH as the equation to convert the applied potential to the RHE scale.^[^ ^23^ ^]^ Cyclic voltammetry (CV) and linear scanning voltammetry (LSV) were employed to test the electrochemical activity of the selected representative standards (The scan rate was 5 mV s^−1^). The double‐layer capacitance (*C*_dl_) was calculated by combining test scan rates of 10–100 mV s^−1^ with CV curves at 0.50 V vs RHE and Δ*j*/2 = (*j*_a_−*j*_c_)/2. The double‐layer capacitance *C*_dl_ was equivalent to the linear slope obtained from the above data, which was proportional to the ECSA of the catalyst. The ECSA was calculated for each sample by the equation ECSA = *C*_dl_/*C*_s_, where the specific capacitance of the sample or the capacitance of the atomically smooth plane of the material per unit area under the same electrolyte conditions was defined as *C*_s_, and the value of *C*_s_ was set to 0.029 mF cm^−2^ as reported in the relevant literature.^[^ ^24^ ^]^ ECSA normalized CO current density: *j*_CO (ECSA)_ = *j*_CO_ × *C*_s_/*C*_dl_, where *j*_CO (ECSA)_ is the ECSA normalized CO sub‐current density, and *j*_CO_ is the average of the sub‐current density of CO. Electrochemical impedance spectroscopy (EIS) measurements were conducted in the frequency range of 1 Hz to 100 KHz, with the ideal potential of −1.3 V versus RHE. The catalyst's geometric area was utilized to compute the current density, and 0.1 m KHCO_3_ solution with a pH of 6.8 was employed as the electrolyte.

### Analysis of Products

The process of CO_2_RR had been carried out in a CO_2_‐saturated KHCO_3_ solution (0.1 m). The potential for electrochemical carbon dioxide reduction ranged from −0.8 to −1.7 V versus RHE with a potential interval of 0.1 V. The composition of the gaseous products was analyzed using the gas chromatograph online with a time interval of 10 min^−1^. Hydrogen (H_2_) was detected by the thermal conductivity detector of the gas chromatograph and carbon monoxide (CO) was detected by the flame ionization detector. The FE calculations for the gas‐phase products (CO and H_2_) are as follows:

(4) $$FE\;=\frac{Q_{\mathrm{CO}}}{Q_{\mathrm{total}}}\;\times 100\%\;=\frac{y\times v\times N\times F}{I\times 60\;s\;\;\min^{-1}\times 24000\;{\mathrm{cm}}^{3}\;{\mathrm{mol}}^{-1}}\;\times 100\%$$

where the concentration (*y*) of CO (or H_2_) in a 1 mL sampling ring in the gas the chromatograph is detected by gas chromatography; the CO_2_ flow rate (*ν*) is 10 mL min^−1^; the number of electron transfers (*N*) to produce 1 mol of CO (or H_2_) is 2; the Faraday constant (*F*) is 96485 C mol^−1^, and the current (*I*) recorded during electrolysis is measured by an electrochemical workstation.^[^ ^25^ ^]^ The liquid product was characterized by nuclear magnetic resonance (NMR) spectroscopy.

### Fabrication ZCB

A polished zinc plate was used as the anode and the three‐phase heterojunction Cu/Cu_2_O‐Sb_2_O_3_‐15 electrocatalyst as the cathode electrode to create the ZCB, which had two separate cavities that were separated by a bipolar film.^[^ ^26^ ^]^ Bipolar membrane, a specific type of ion exchange membrane with an anion exchange layer and a cation exchange layer, was employed in this study. After the application of direct current, water molecules will produce protons (H^+^) and hydroxide ions (OH^−^) after splitting inside the membrane. The cathodic electrolyte was 1.0 M KHCO_3_ and the anodic electrolyte was 6 m KOH/0.02 m Zn (CH_3_COO)_2_–CO_2_ saturated solution.

### Theoretical Details

All DFT calculations were performed on the Vienna Ab initio Simulation Package. The elementary nuclei and valence electrons were represented by the projection augmentation wave method and the plane wave basis function with a cutoff energy of 400 eV. All calculations were performed using the Perdew‐Burke‐Ernzerh generalized gradient approximation of the exchange‐correlated generalized function. Geometric optimization with force convergence less than 0.05 eV Å^−1^. Calculated free energy curves for CO_2_ formation and hydrogenation side reactions on Cu/Cu_2_O‐Sb_2_O_3_‐15 and Cu/Cu_2_O.

## Conflict of Interest

The authors declare no conflict of interest.

## Supporting information

Supporting Information

## Data Availability

The data that support the findings of this study are available from the corresponding author upon reasonable request.
