# Supplementary material for: Three‐Phase‐Heterojunction Cu/Cu2O–Sb2O3 Catalyst Enables Efficient CO2 Electroreduction to CO and High‐Performance Aqueous Zn–CO2 Battery
Source: Adv Sci (Weinh). 2024 Feb 27;11(16):2306858. doi: 10.1002/advs.202306858 (PMC11040368; doi:10.1002/advs.202306858)
Supplement: Supplementary file 1 — Supporting Information [file ADVS-11-2306858-s001.pdf]

ADVANCED SCIENCE
Open Access

Supporting Information

for *Adv. Sci.*, DOI 10.1002/advs.202306858

Three-Phase-Heterojunction Cu/Cu_2O–Sb_2O_3 Catalyst Enables Efficient CO_2 Electroreduction to CO and High-Performance Aqueous Zn–CO_2 Battery

Junjie Ma, Fang Huang, Aihao Xu, Dong Wei, Xiangyu Chen, Wencan Zhao, Zhengjun Chen, Xucai Yin, Jinliang Zhu, Huibing He and Jing Xu**

Three-Phase-Heterojunction $Cu/Cu_2O-Sb_2O_3$ Catalyst Enables Efficient CO_2 Electroreduction to CO and High-Performance Aqueous Zn-CO_2 Battery

Junjie Ma[a], Fang Huang[a], Aihao Xu[a], Dong Wei[a], Xiangyu Chen[a], Wencan Zhao[a], Zhengjun Chen[a], Xucai Yin [a], Jinliang Zhu[b], Huibing He[a*], Jing Xu[a*]

[a] *School of Chemistry and Chemical Engineering, Guangxi Key Laboratory of Petrochemical Resource Processing and Process Intensification Technology, Guangxi University, Nanning 530004, PR. China*

[b] *School of Resources, Environment, and Materials, Collaborative Innovation Center of Sustainable Energy Materials, Guangxi Key Laboratory of Processing for Non-ferrous Metals and Featured Materials, Guangxi University, Nanning, 530004 P. R. China*

**Corresponding author: Huibing He, E-mail address: huibinghe@gxu.edu.cn*
Jing Xu, E-mail address: xujing@ecust.edu.cn

Supplementary Experimental Section

1. Preparation of the Electrode

The work electrodes were typically prepared as follows: 8 mg catalyst, 1900 μL isopropanol, and 100 μL Nafion solution (5.0 wt%) were mixed and ultrasonicated for one hour until a homogeneous ink was obtained. Subsequently, the ink was evenly spread over the carbon paper with a catalyst loading of ~1 mg cm^{-2}.

2. Evaluation of TOF

The turn over frequency (TOF) for CO was calculated as follows:

$$TOF(h^{-1})=\frac{j_{CO}\times S/(NF)}{m_{cat}\times w/M_{Cu}}\times 3600$$

where j_{CO} is the partial current density for CO production (A cm^{-2}), S is the surface area of working electrode (1×1 cm^2), n is the number of electron transferred for product formation (n=2), F is Faraday constant (96485 C mol^{-1}), m_{cat} is the catalyst mass in the electrode (g), *w* is Cu loading in the catalyst, and M_{Cu} is the atomic mass of Cu (63.5 g·mol^{-1}).

3. Calculation of the Theoretical Potential of the Aqueous Rechargeable ZCB [1]

The half-reaction (CO_2RR) that took place on the electrode during the discharging process: (All applied potentials were relative to the standard hydrogen electrode (SHE))

Cathode (1M $KHCO_3$ sat. CO_2, pH = 8.1):

$$CO_2+2H^++2e^-\rightarrow CO+H_2O$$

$$E^{\theta}_{CO_2/CO}=-0.106V\ vs.\ SHE$$

Anode (6 M KOH with 0.02 M Zn $(CH_3COO)_2$):

$$Zn-2e^-\rightarrow Zn^{2+}$$

$$Zn^{2+}+4OH^-\rightarrow Zn(OH)_4^{2-}$$

$E^{\theta}_{Zn(OH)_4^{2-}/Zn}$=-1.199V vs.SHE

Overall reaction during discharge:

$$Zn+CO_2+2H^{+}+4OH^{-}\rightarrow Zn(OH)_4^{2-}+CO+H_2O$$

$$E_c=E^{\theta}_{CO_2/CO}-\frac{RT}{nF}ln\left[\frac{1}{\alpha^2_{H^+}}\right]=-0.106-\frac{0.0592}{2}lg\left[\frac{1}{(10^{-8.1})^2}\right]V=-0.585V$$

$$E_a=E^{\theta}_{Zn(OH)_4^{2-}/Zn}-\frac{RT}{nF}ln\left[\frac{\alpha_{Zn}\times\alpha^4_{OH^-}}{\alpha_{Zn(OH)_4^{2-}}}\right]=-1.199-\frac{8.314\times 298.15}{2\times 96485}ln\left[\frac{6^4}{0.02}\right]V=-1.342V$$

The whole process of battery discharge can be described as:

$E_{discharge}=E_c - E_a$= -0.585- (-1.342) V= 0.757 V

The energy density (ED) of the Zn-CO_2 electrochemical battery:

Zn theoretical capacity under standard conditions: C_{Zn}=825 mAh g^{-1}

$ED_{Zn}=C_{Zn}\times E_{tho}$=825 mAh g^{-1}×0.757V=624.5 Wh Kg^{-1}

The half-reaction that takes place on the electrode during the charging process: (All applied potentials were relative to the SHE)

Cathode (1M $KHCO_3$ sat. CO_2, pH = 8.1):

$$H_2O\rightarrow\frac{1}{2}O_2+2H^{+}+2e^{-}$$

$$E^{\theta}_{H_2O/O_2}=1.229V\ vs.SHE$$

$$E_c=E^{\theta}_{H_2O/O_2}-\frac{RT}{nF}ln\left[\frac{1}{\alpha^2_{H^+}}\right]=1.229-\frac{0.0592}{2}lg\left[\frac{1}{(10^{-8.1})^2}\right]V=0.75V$$

Anode (6 M KOH with 0.02 M Zn $(CH_3COO)_2$):

$$Zn(OH)_4^{2-}\rightarrow Zn^{2+}+4OH^{-}$$

$$Zn^{2+}+2e^{-}\rightarrow Zn$$

Overall reaction during charge:

$$Zn(OH)_4^{2-}+H_2O\rightarrow Zn+\frac{1}{2}O_2+2H^{+}+4OH^{-}$$

$E_{charge}= E_c - E_a$= 0.75− (−1.342) V= 2.092 V

T=298.15 K

n=2 (the number of electrons transferred per mole of product)

F=96485 C mol^{-1}(Faradaic constant) and α is the corresponding activity

R=8.314 J K^{-1} mol^{-1} (molar gas constant)

Supplementary Figures

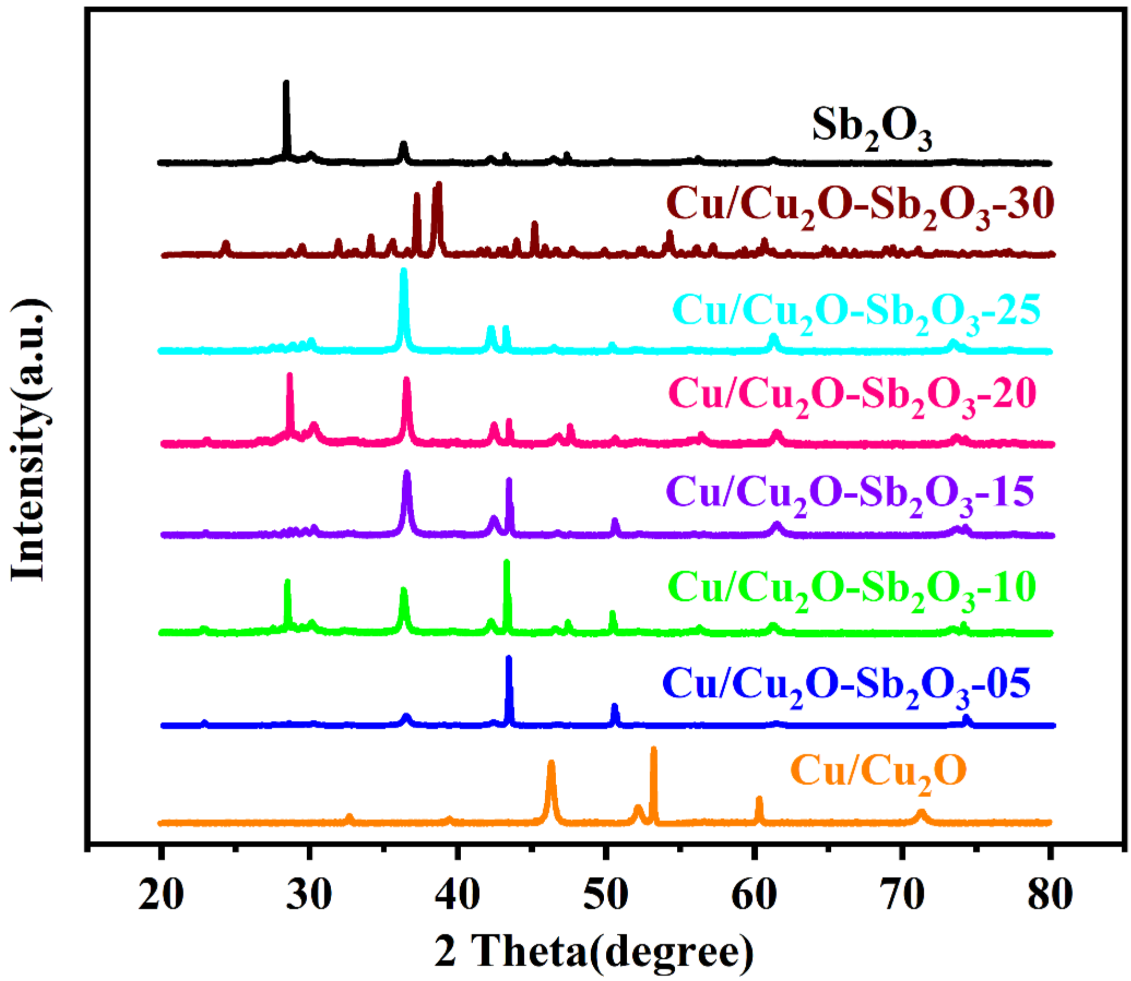

Figure S1. XRD patterns of Cu/Cu_2O, Cu/Cu_2O-Sb_2O_3-5, Cu/Cu_2O-Sb_2O_3-10, Cu/Cu_2O-Sb_2O_3-15, Cu/Cu_2O-Sb_2O_3-20, Cu/Cu_2O-Sb_2O_3-25, Cu/Cu_2O-Sb_2O_3-30 and Sb_2O_3.

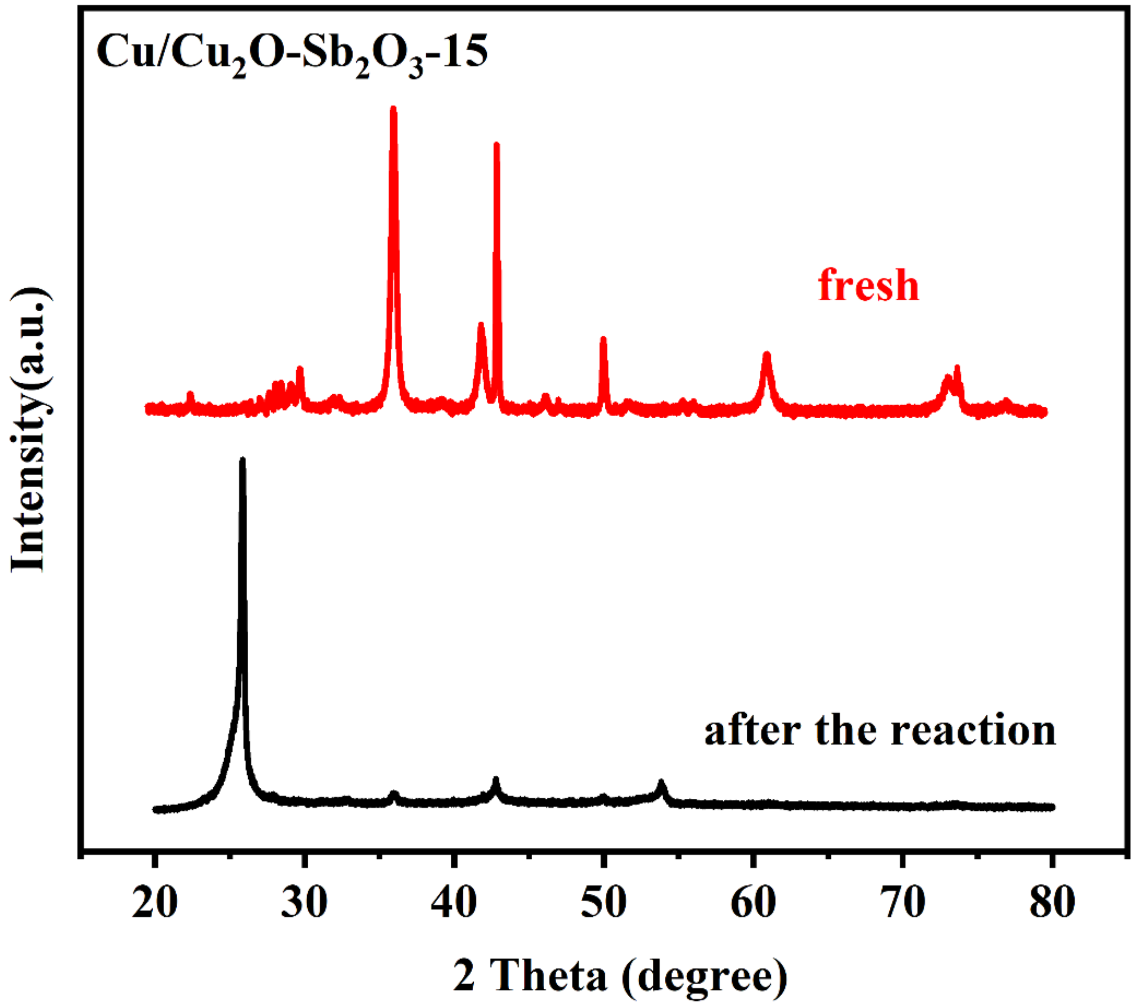

Figure S2. XRD patterns of fresh and used (i.e., after 36 h CO_2 reduction electrolysis) $Cu/Cu_2O-Sb_2O_3-15$ catalyst.

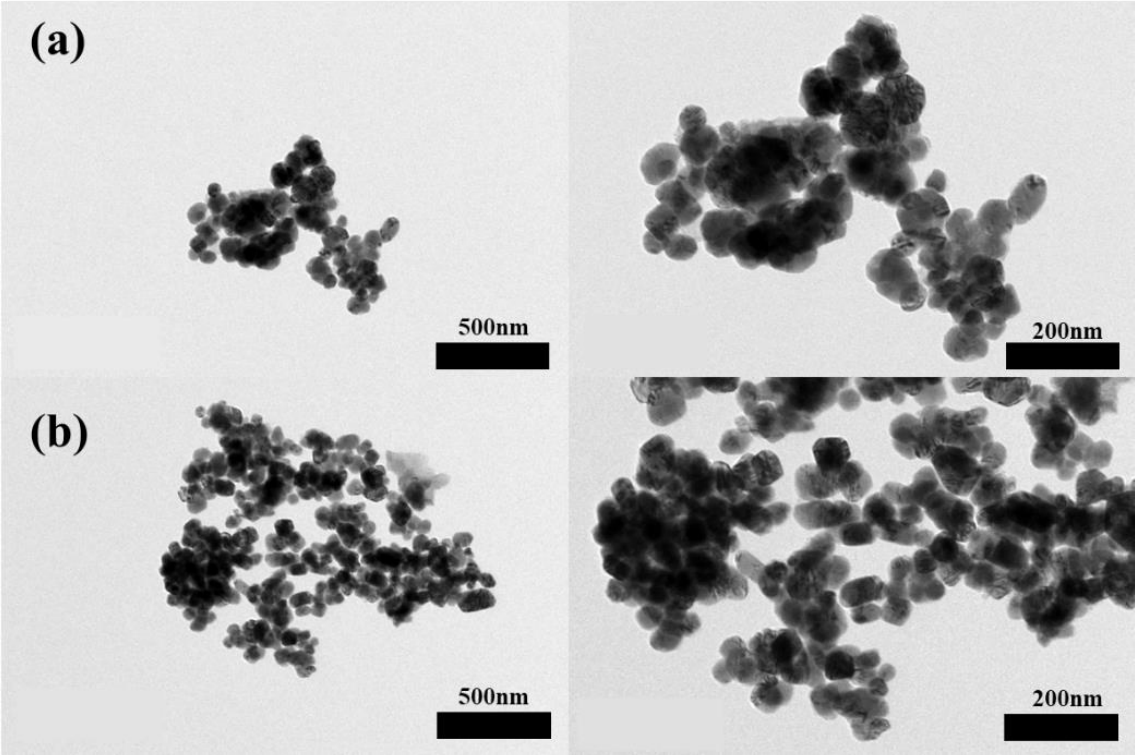

Figure S3. Low-and high-magnification TEM image of (a) $Cu/Cu_2O-Sb_2O_3$-15 and (b) Cu/Cu_2O.

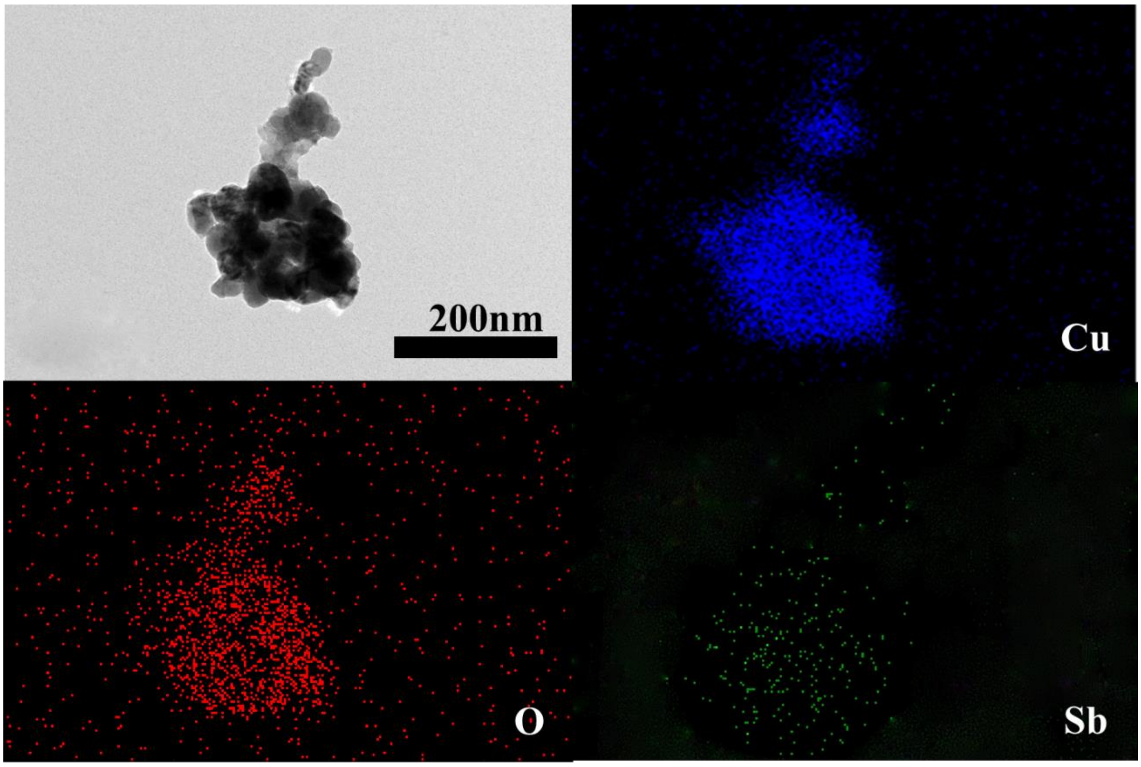

Figure S4. TEM image of Cu/Cu_2O-Sb_2O_3-15 and the corresponding EDS mapping images of copper (blue), oxygen (red), and antimony (green) elements in Cu/Cu_2O-Sb_2O_3-15.

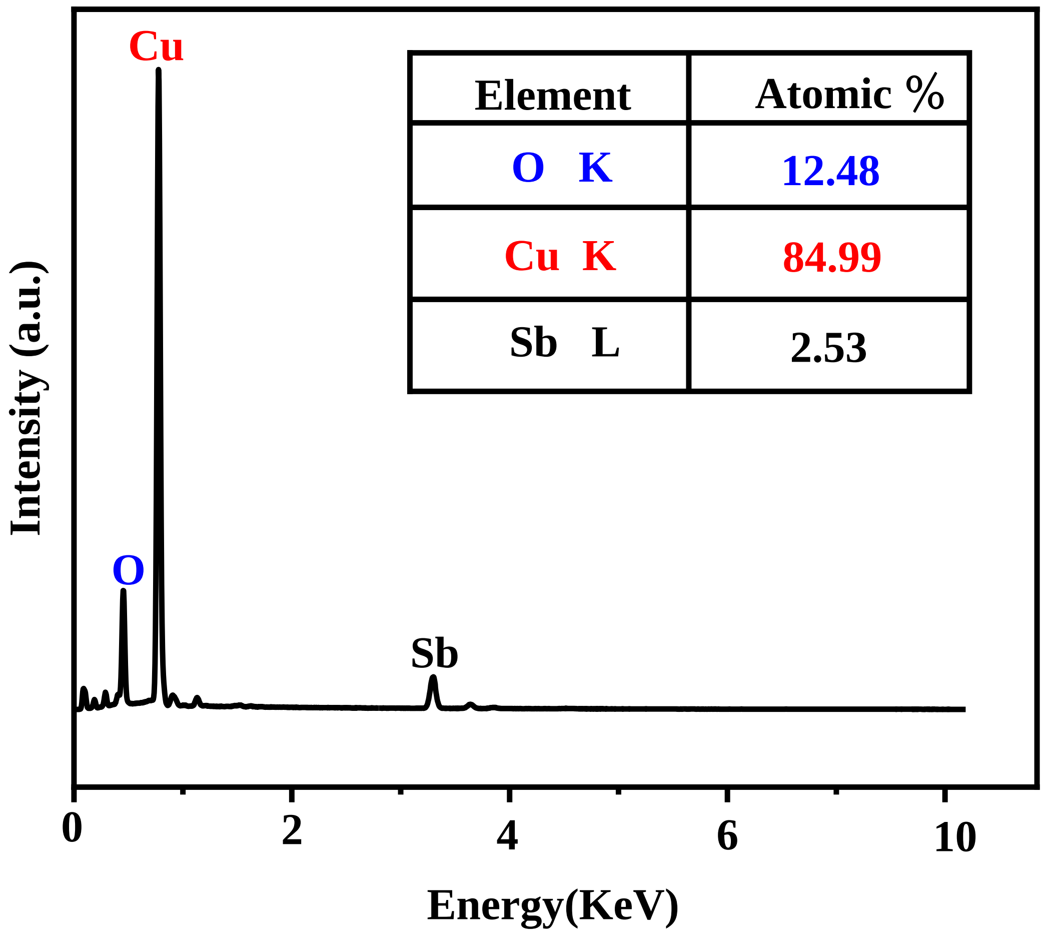

Figure S5. EDS spectrum of Cu/Cu_2O-Sb_2O_3-15 collected in the whole TEM region (**Figure S4**).

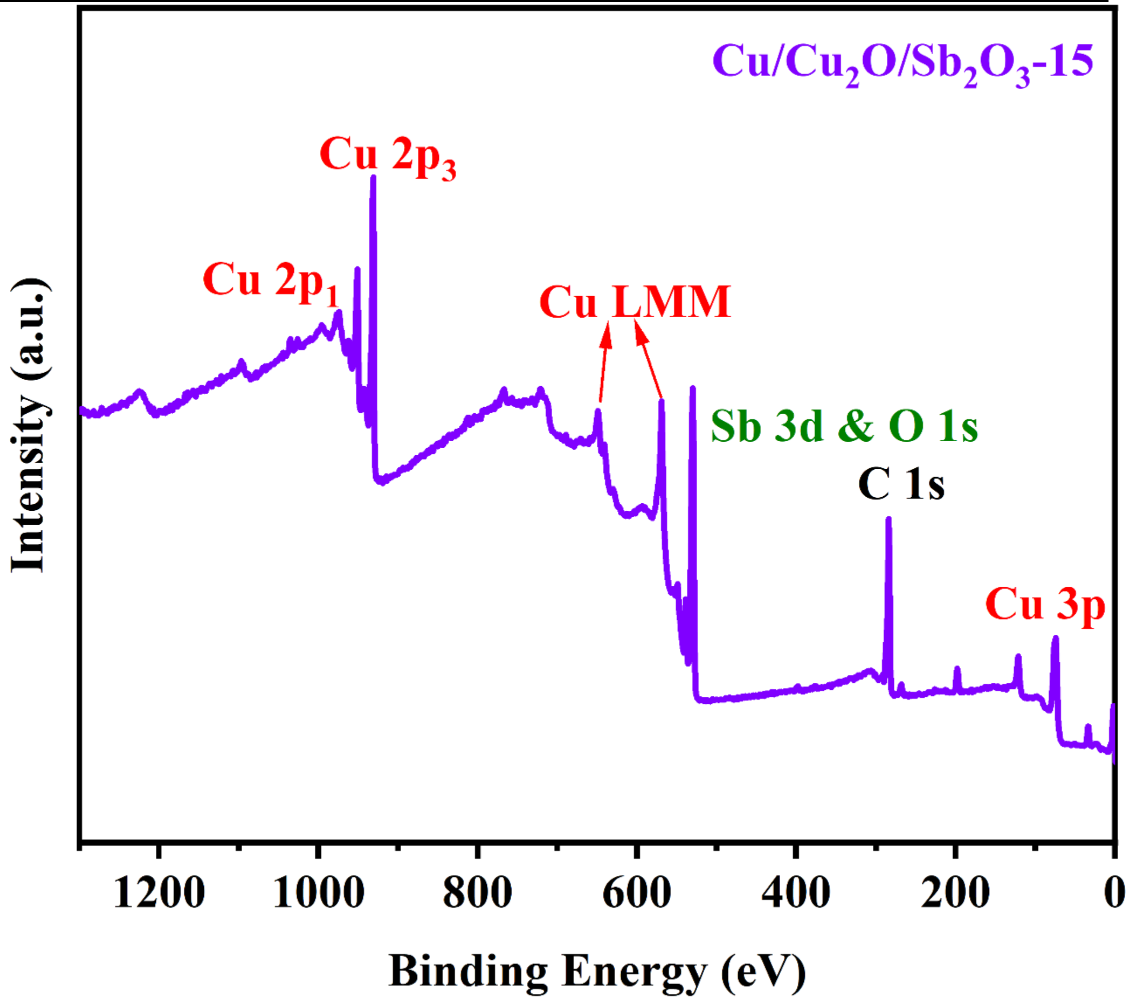

Figure S6. Survey XPS spectra of Cu/Cu_2O-Sb_2O_3-15 catalyst.

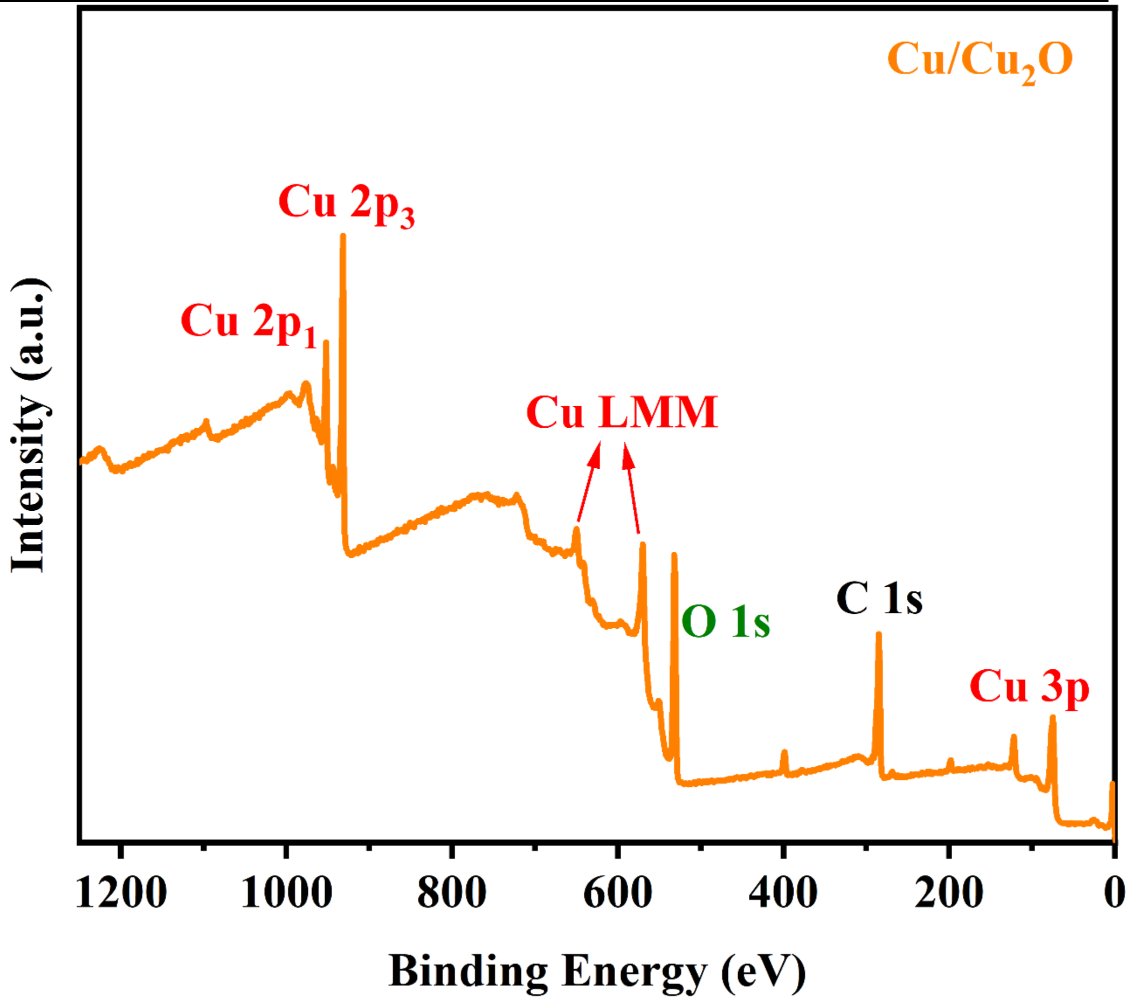

Figure S7. Survey XPS spectra of Cu/Cu_2O catalyst.

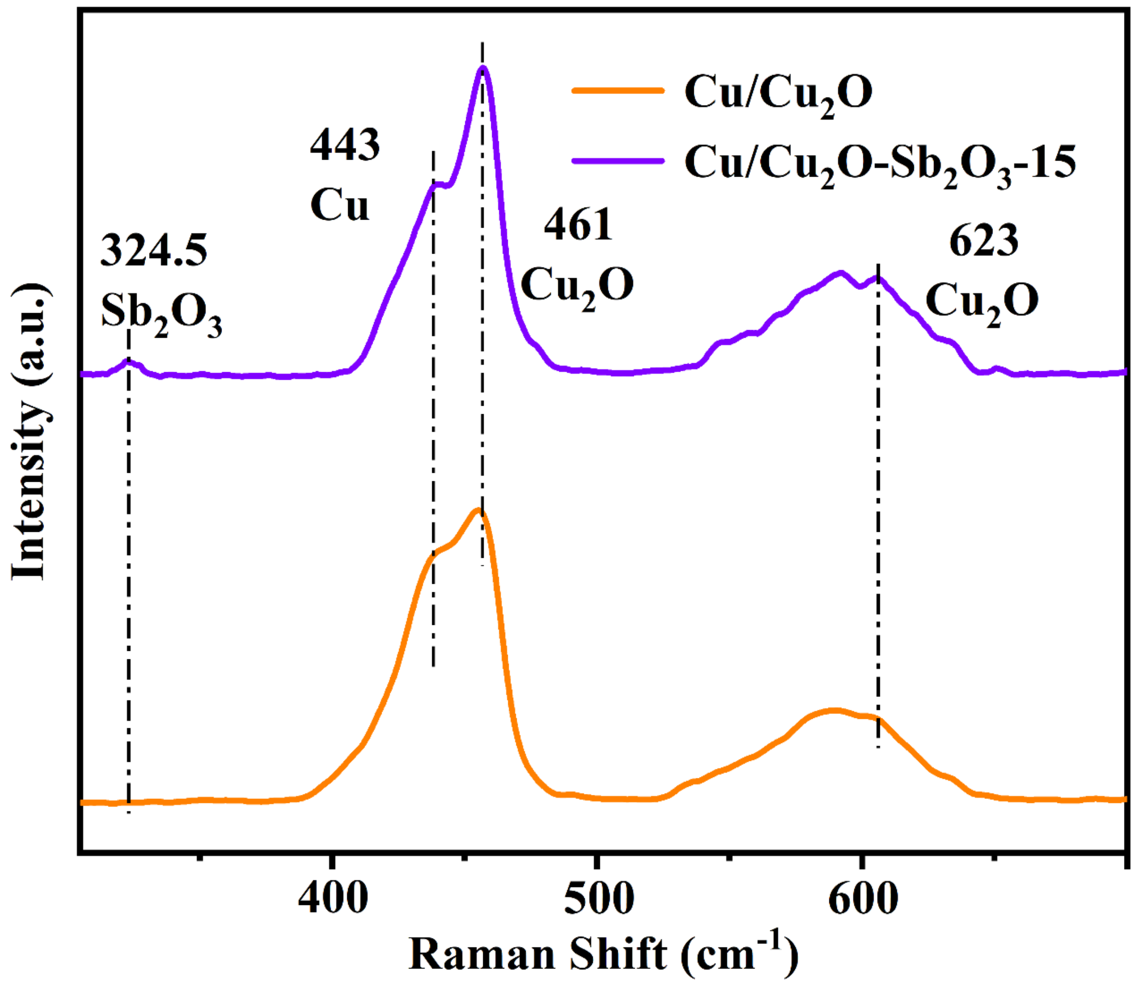

Figure S8. Raman spectra graph of Cu/Cu_2O-Sb_2O_3-15 versus Cu/Cu_2O.

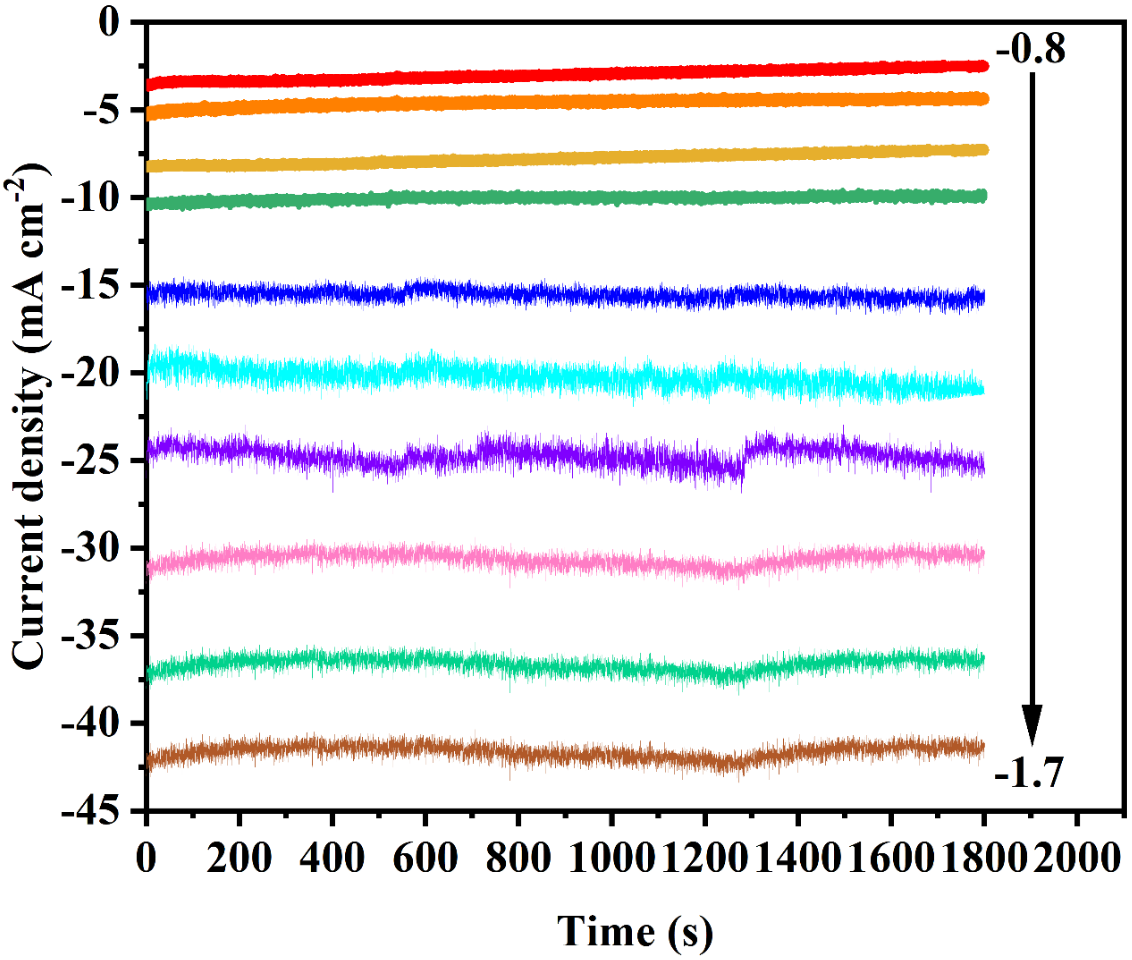

Figure S9. I-T curves of $Cu/Cu_2O-Sb_2O_3$-15 at different applied potentials (*vs.* RHE).

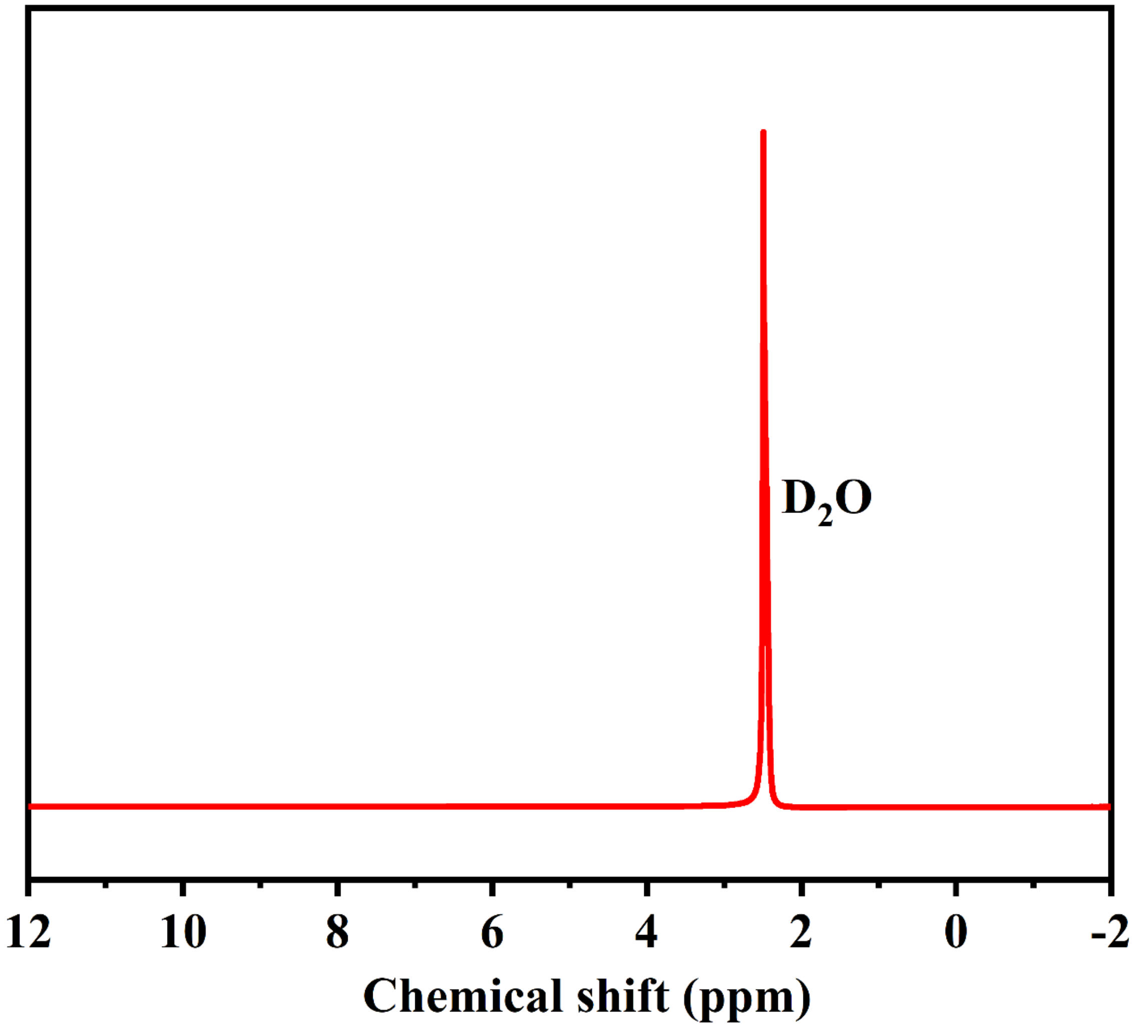

Figure S10. ^{1}H NMR result for the liquid products of the CO_2RR for Cu/Cu_2O-Sb_2O_3-15 electrode at a potential of -1.3 V *vs.* RHE.

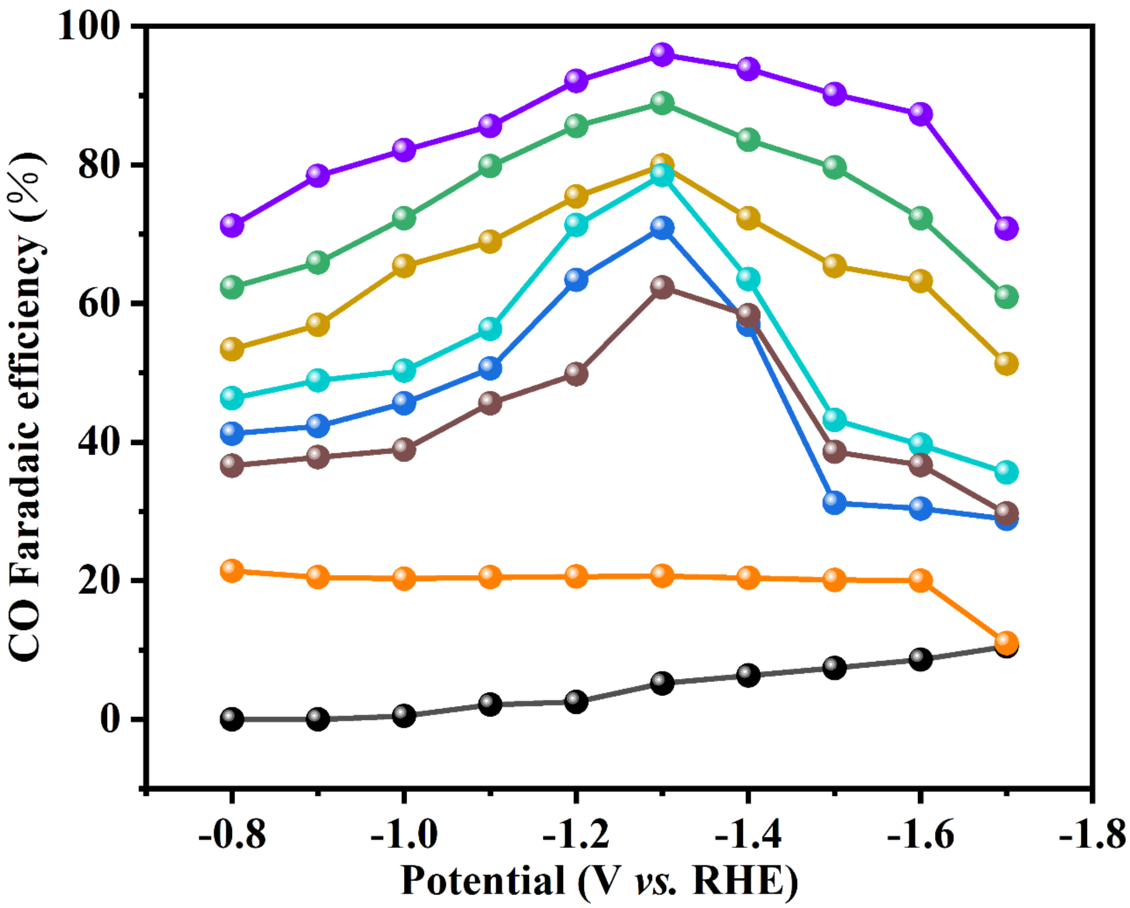

Figure S11. Comparison of CO faradaic efficiency of $Cu/Cu_2O-Sb_2O_3$ series catalysts with different chemical compositions (icons are shown below).

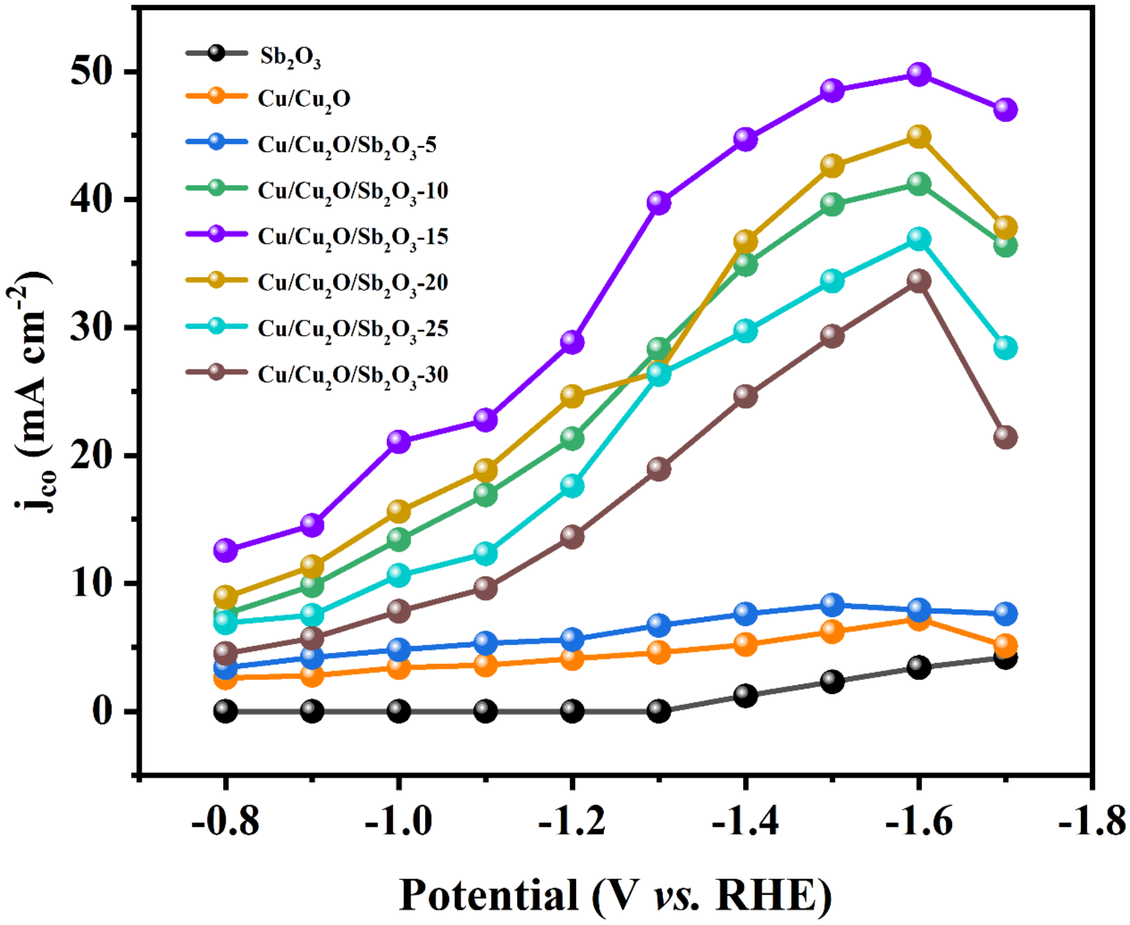

Figure S12. Comparison of the partial current density of Cu/Cu_2O-Sb_2O_3 series catalysts with different chemical compositions.

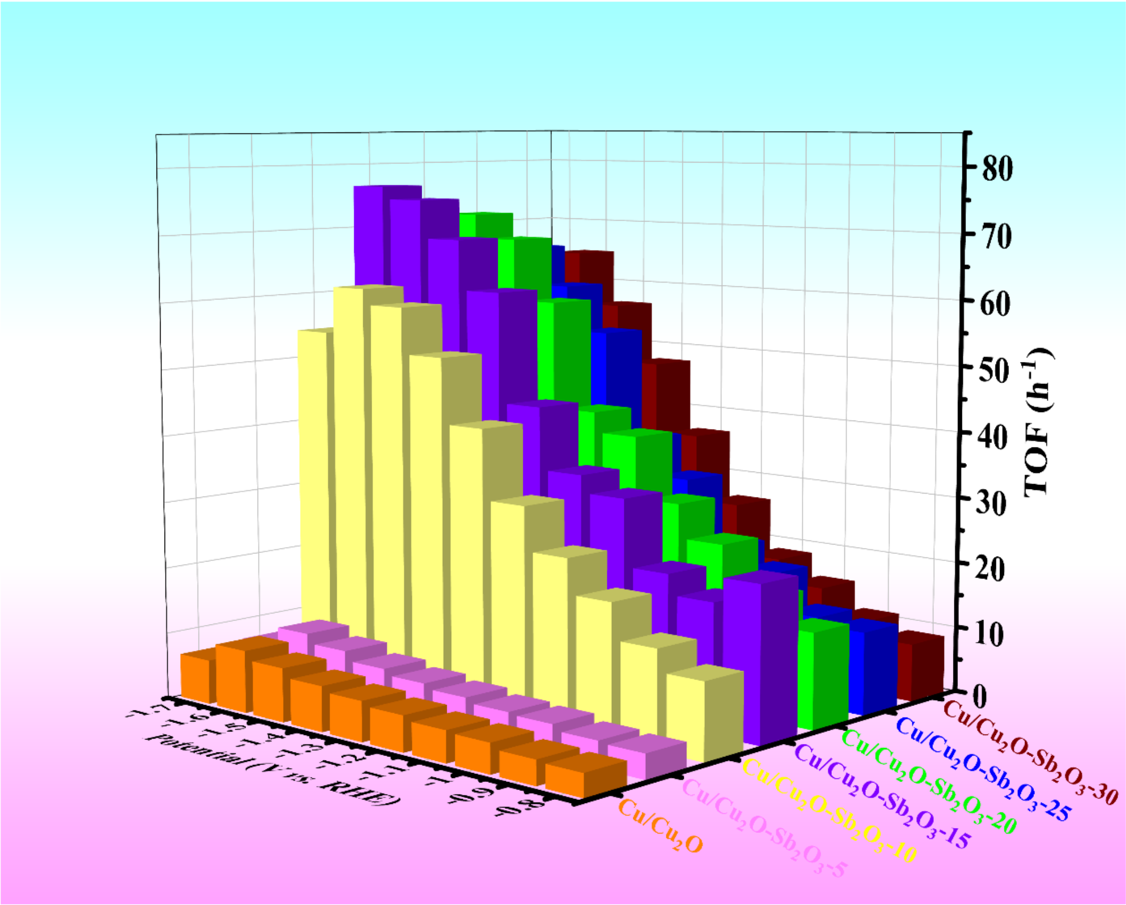

Figure S13. Calculated TOF of $Cu/Cu_2O-Sb_2O_3$ series catalysts.

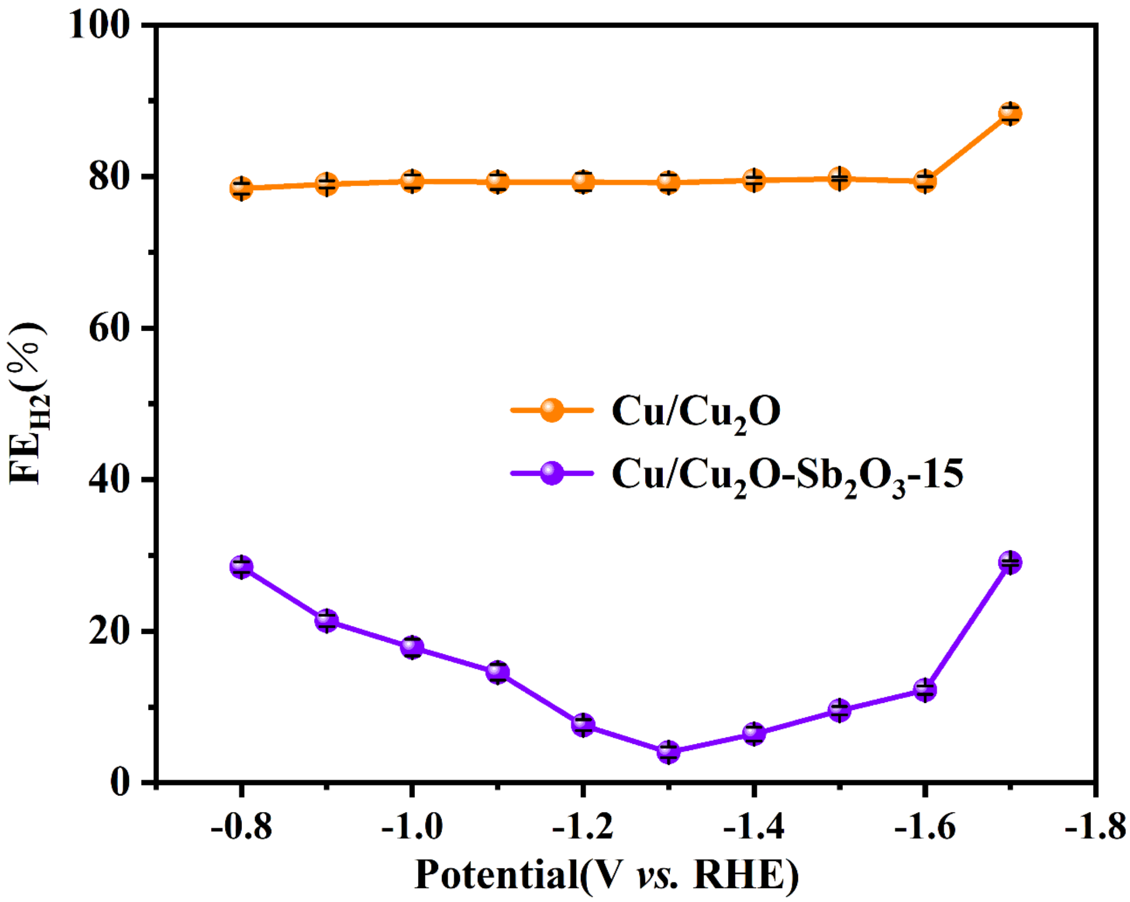

Figure S14. FEs of H_2 for the main electrodes at different potentials.

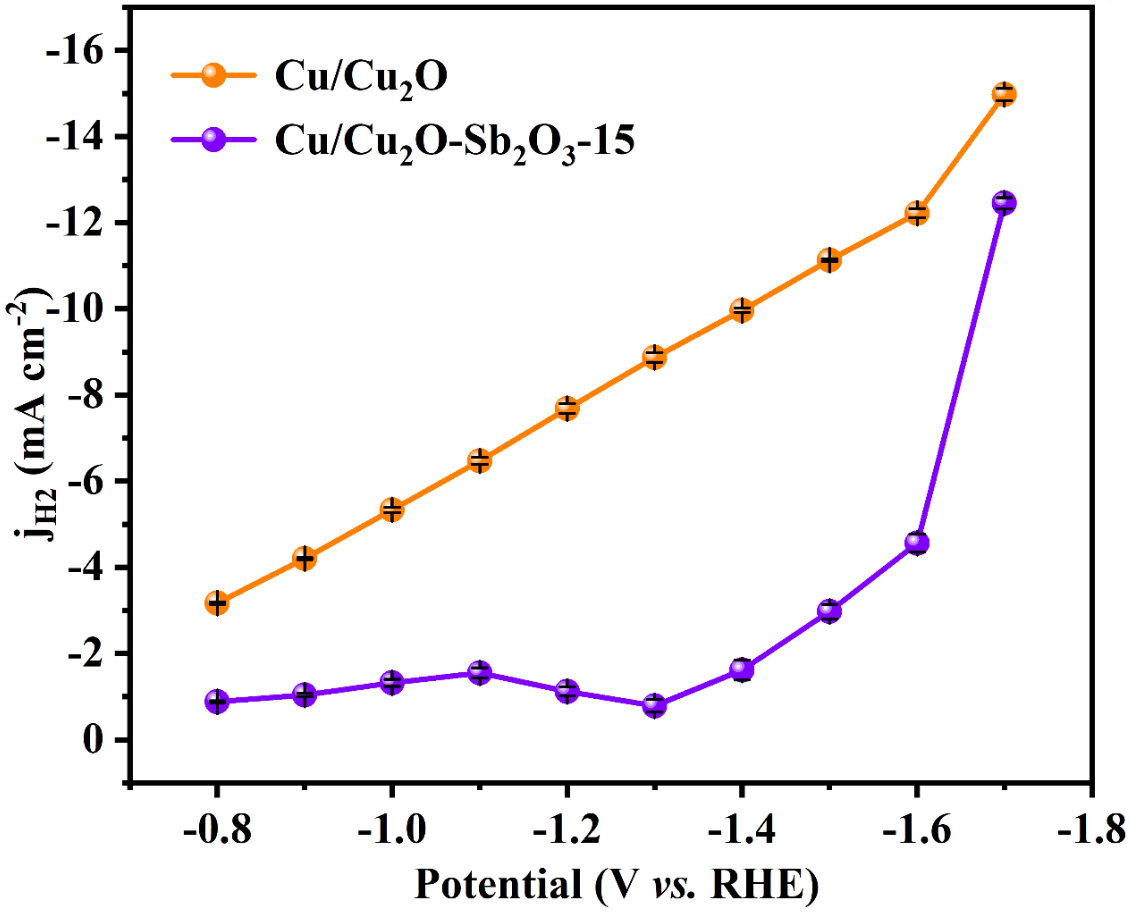

Figure S15. Partial current density of H_2 for the main electrodes at different potentials.

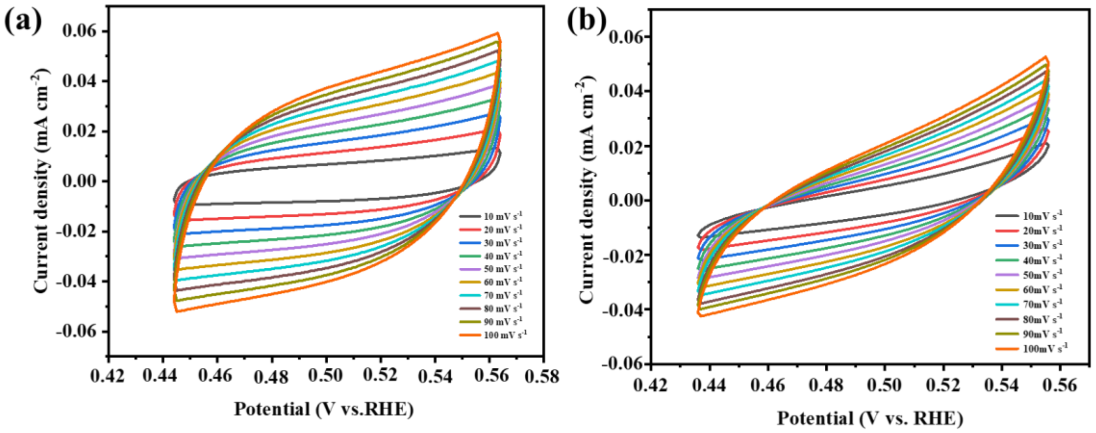

Figure S16. Electrochemical surface area measurement. Cyclic voltammetry scans on (a) Cu/Cu_2O-Sb_2O_3-15, (b) Cu/Cu_2O between 0.42 and 0.58 V in CO_2-saturated 0.1 M $KHCO_3$ solution at scan rates of 10, 20, 30, 40, 50, 60, 70, 80, 90 and 100 mV s^{-1}.

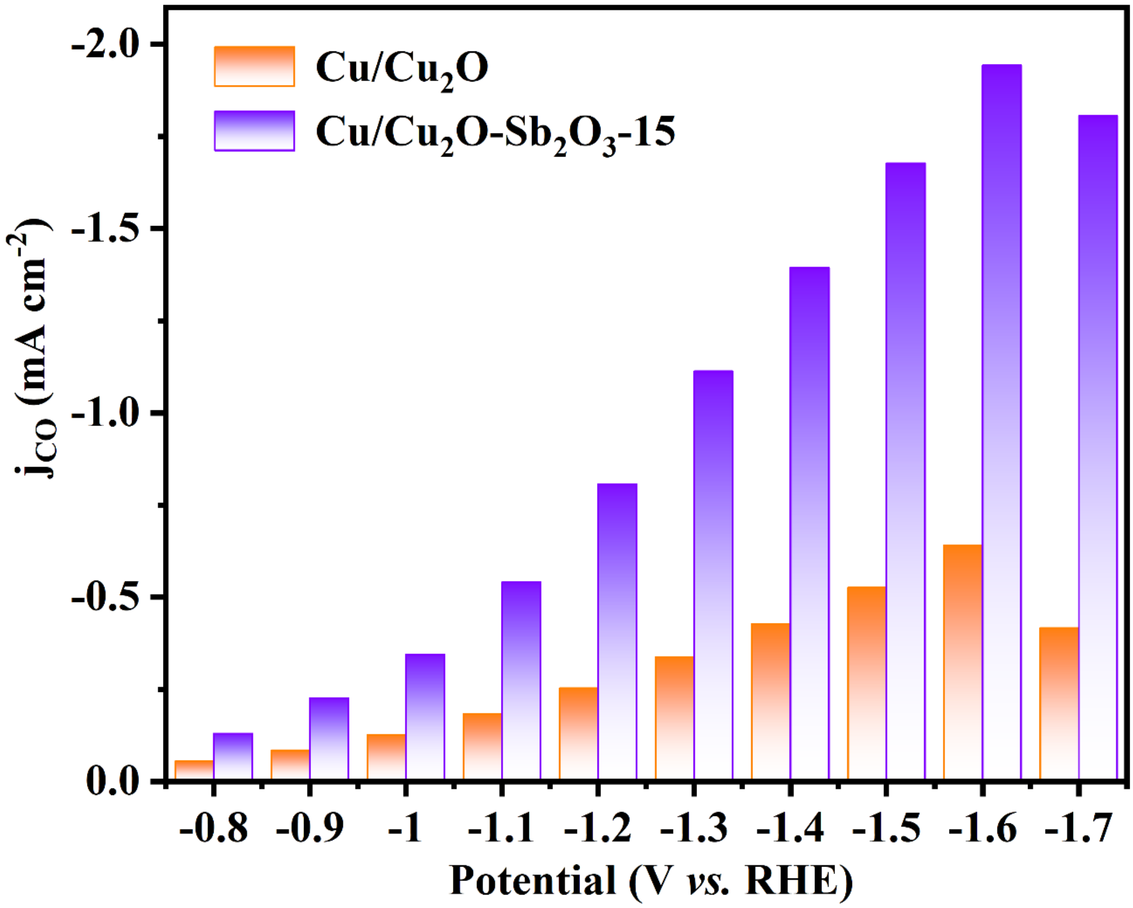

Figure S17. ECSA-normalized j_{CO} on Cu/Cu_2O and $Cu/Cu_2O-Sb_2O_3-15$.

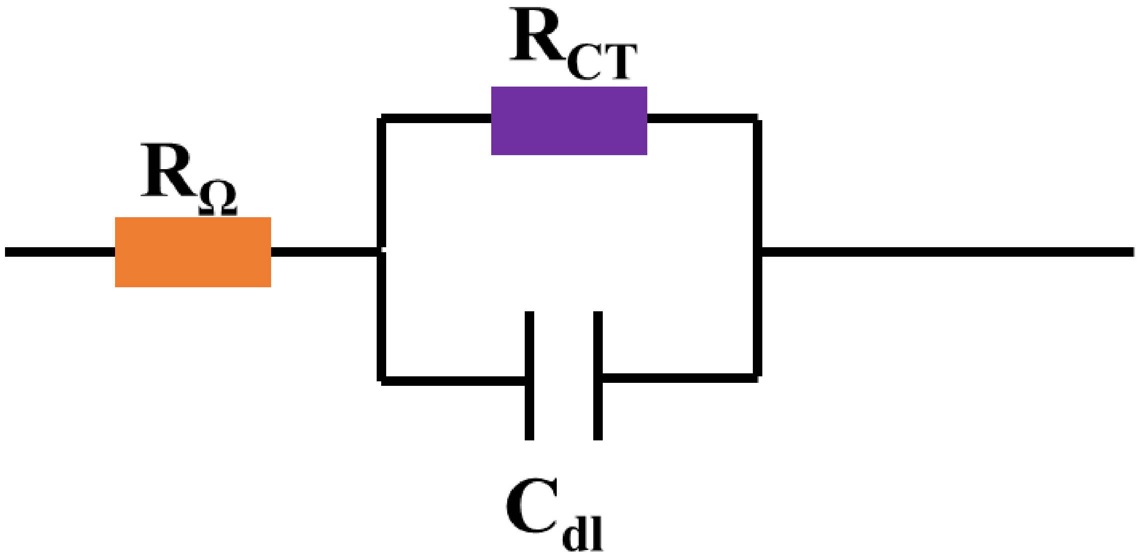

Figure S18. Figure (3e) corresponded to the equivalent circuit.

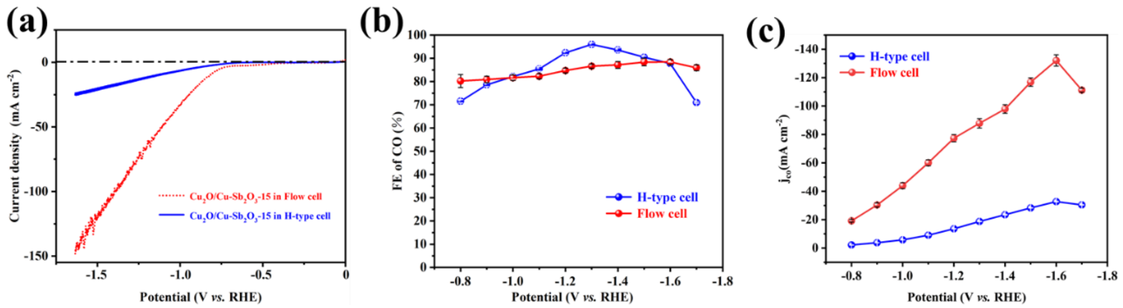

Figure S19. (a) LSV curve of Cu/Cu_2O-Sb_2O_3-15 in H-type cell and flow cell at a scan rate of 5 mV s^{-1}. (b) Faradaic efficiency (c) Partial current density of CO for Cu/Cu_2O-Sb_2O_3-15 in H-type cell and flow cell. (A flow cell equipped with a GDE device was applied for CO_2RR. A commercial Pt electrode was used as an anode and an Ag/AgCl acted as the reference electrode. 1 M KOH aqueous solution was utilized as electrolytes, which were separated by a piece of anion-exchange membrane.

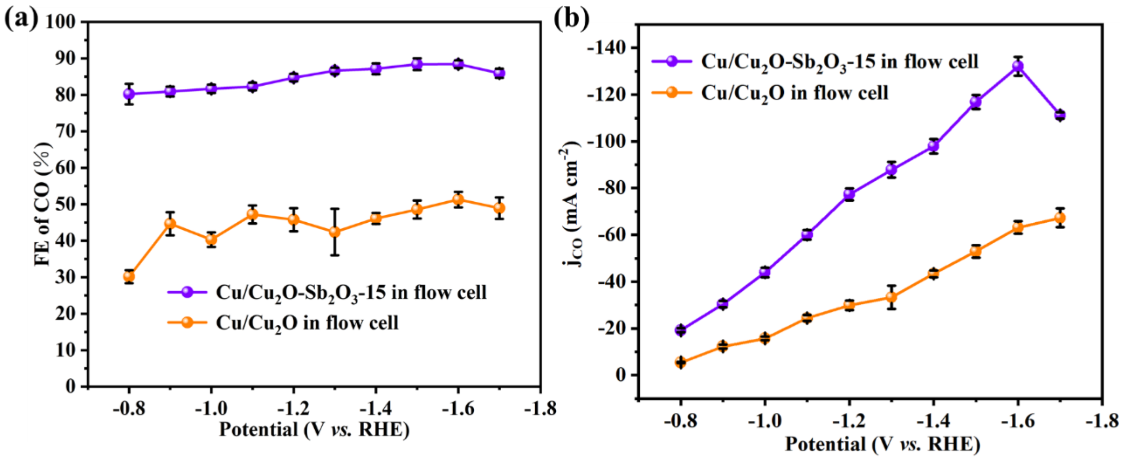

Figure S20. (a) Faradaic efficiency (b) Partial current density of CO for Cu/Cu_2O and Cu/Cu_2O-Sb_2O_3-15 in flow cell.

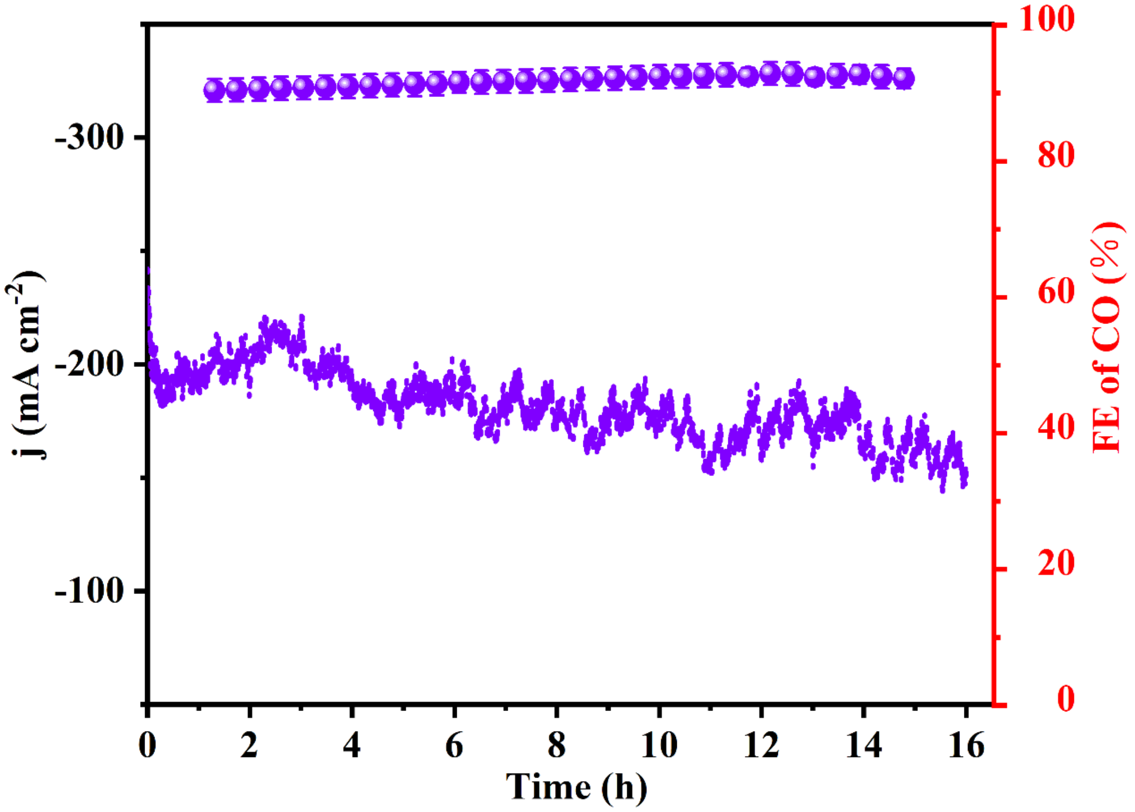

Figure S21. Long-term electrolysis at −1.3V *vs.* RHE in flow cell. The electrolyte tested is 1.0 M KOH. The loading of Cu/Cu_2O-Sb_2O_3-15 is 1.0 mg cm^{-2}.

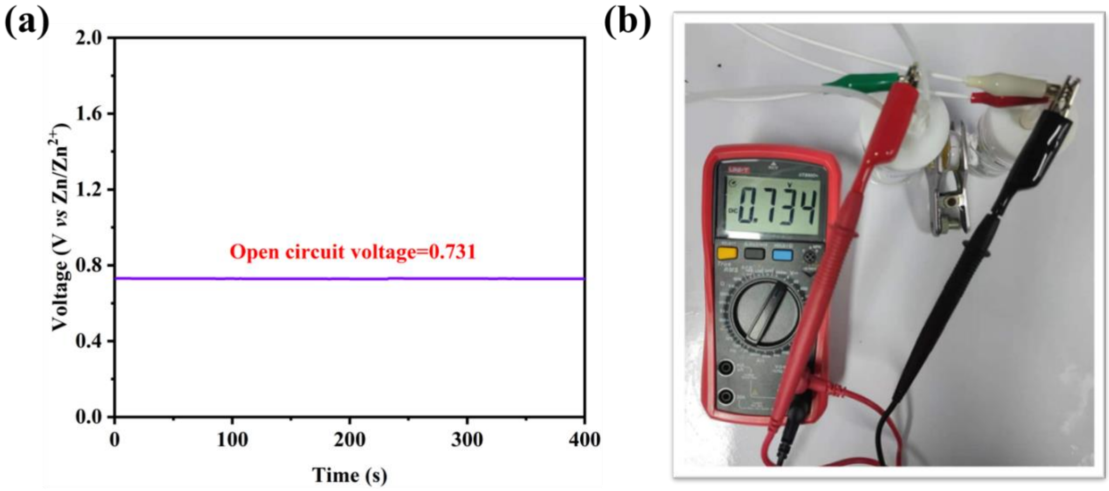

Figure S22. The open circuit voltage (OCV) of the reversible aqueous ZCB.

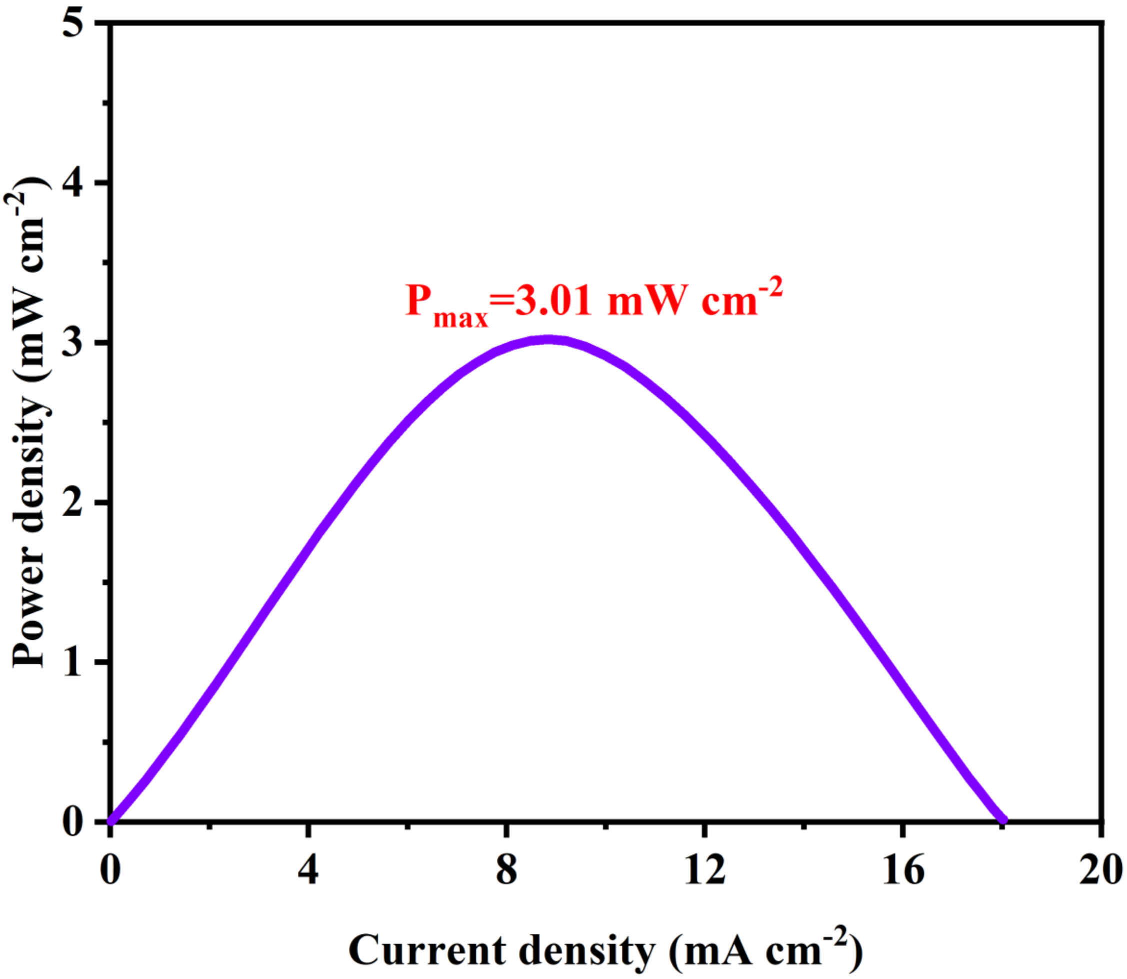

Figure S23. Power density curves of the reversible aqueous ZCB with $Cu/Cu_2O-Sb_2O_3$-15 cathode.

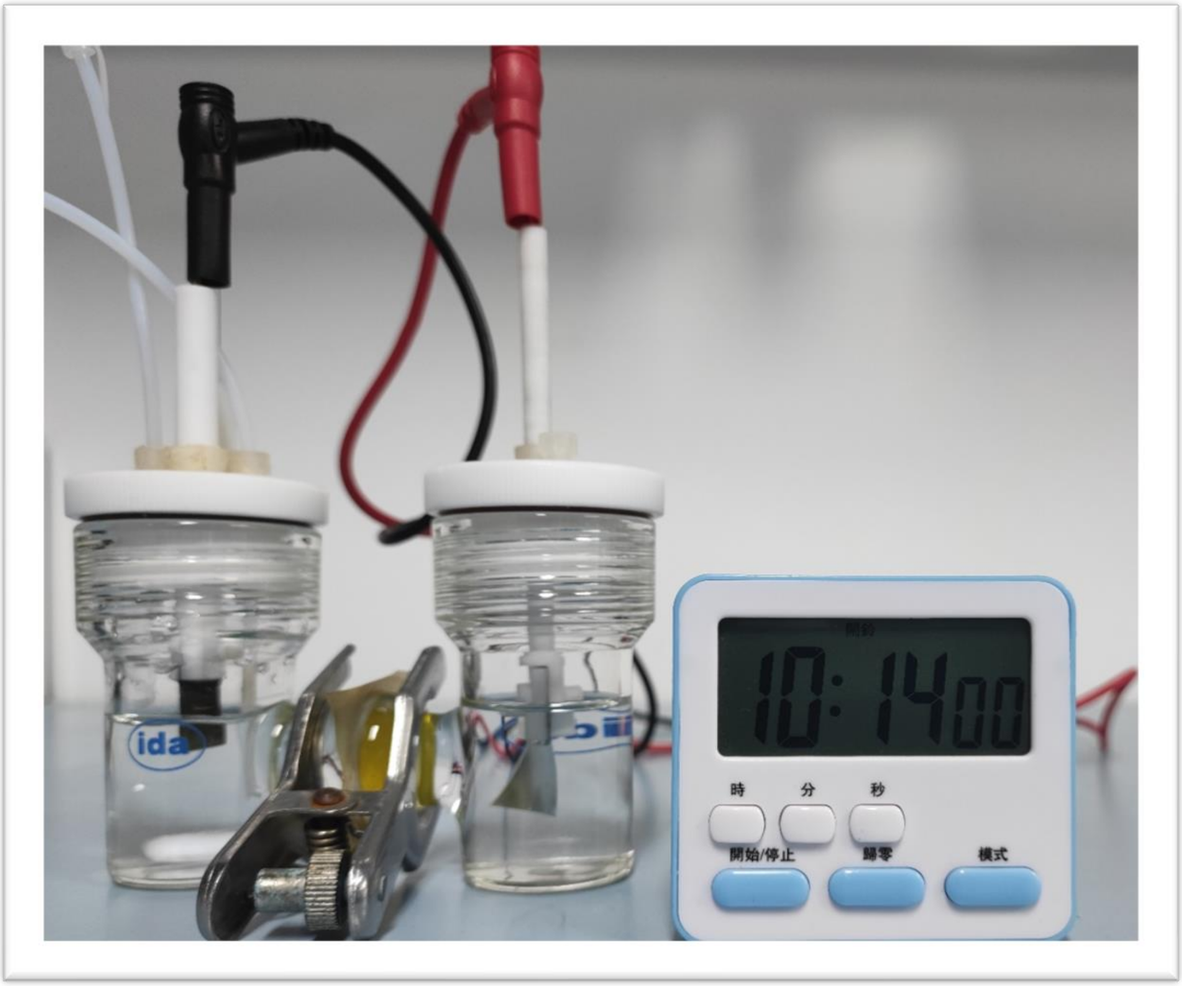

Figure S24. Optical image of an electronic clock powered by the reversible aqueous ZCB with Cu/Cu_2O-Sb_2O_3-15 cathode.

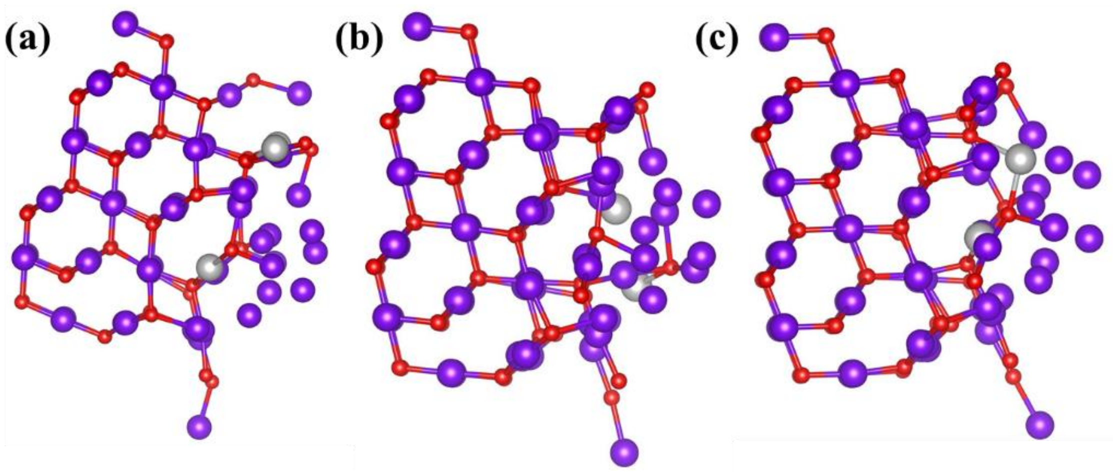

Figure S25. Schematic structures of the three possible Cu/Cu_2O-Sb_2O_3-15 nano-catalyst.

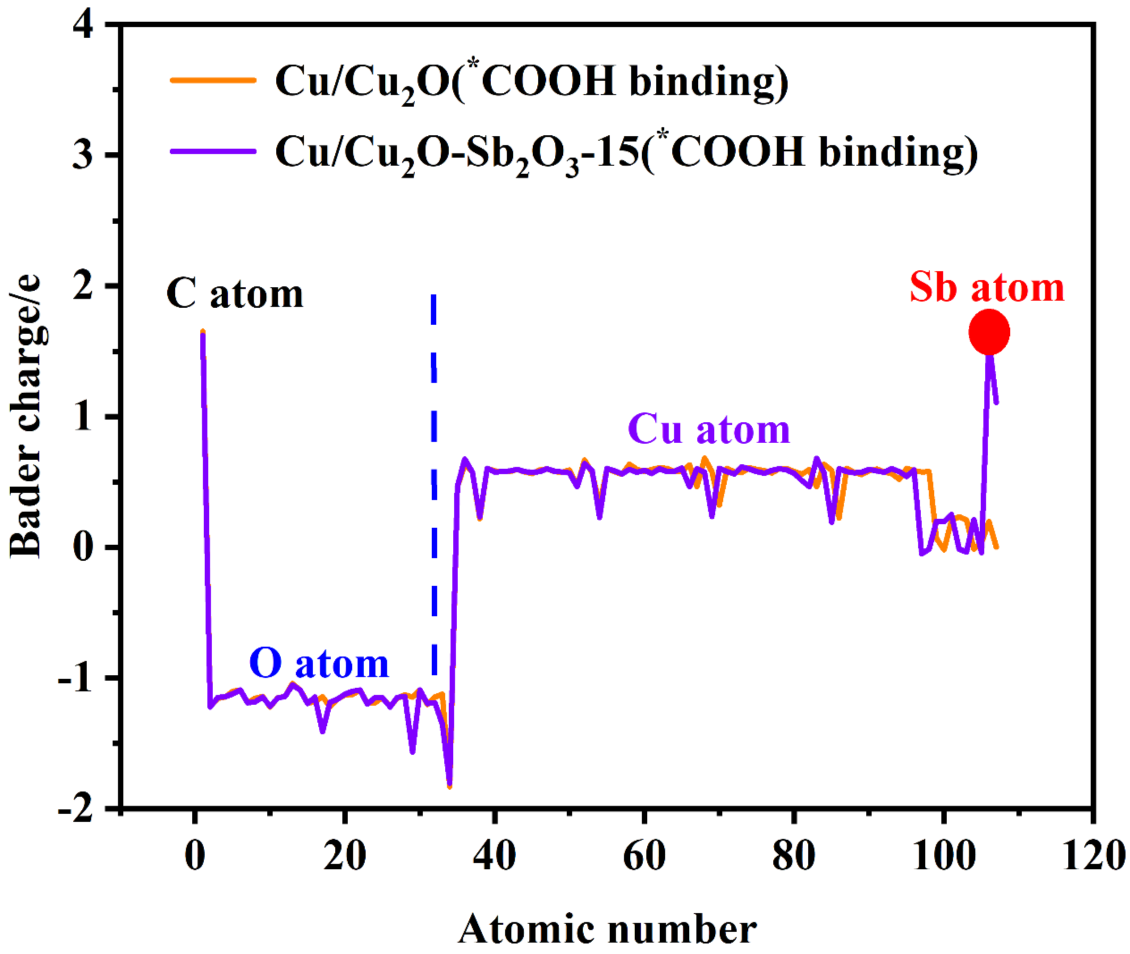

Figure S26. Bader charges racking after $^{*}COOH$ binding on Cu/Cu_2O and Cu/Cu_2O-Sb_2O_3-15.

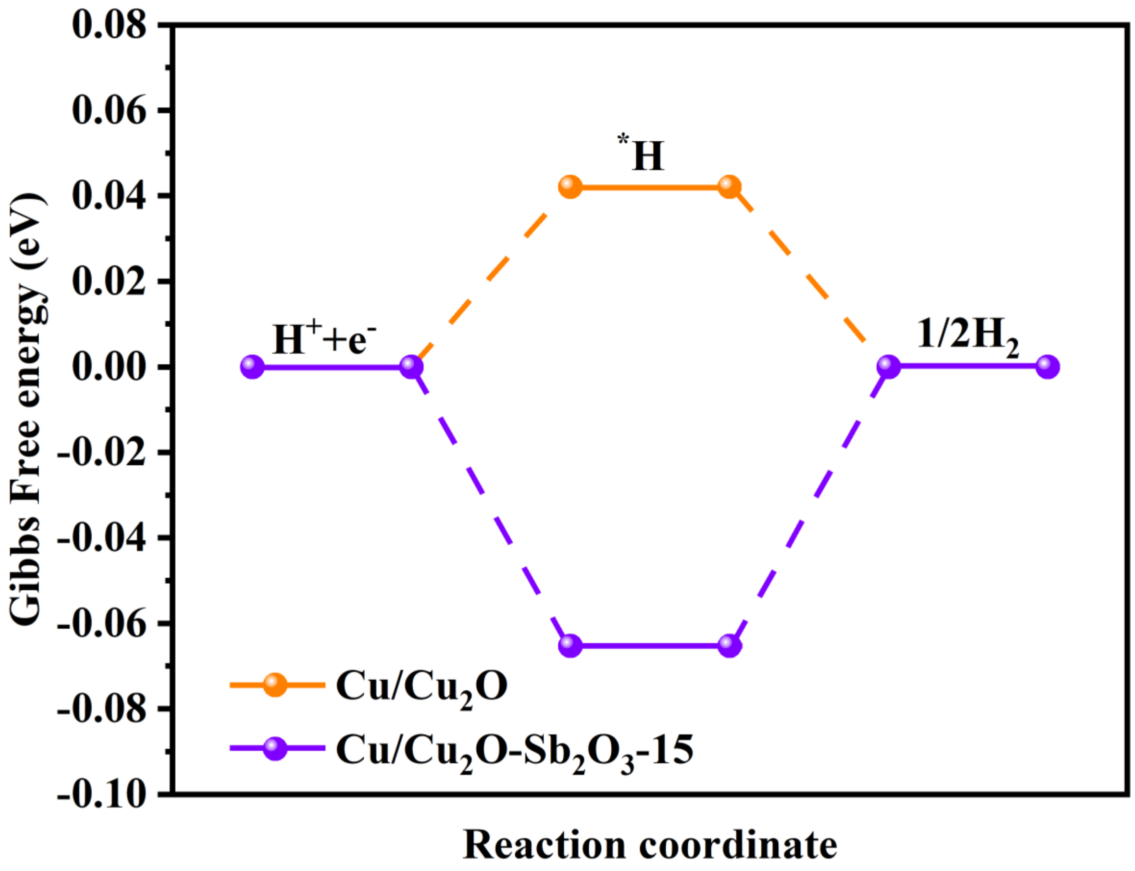

Figure S27. Free-energy energy-barrier diagrams of the side reactions HER on Cu/Cu_2O and Cu/Cu_2O-Sb_2O_3-15.

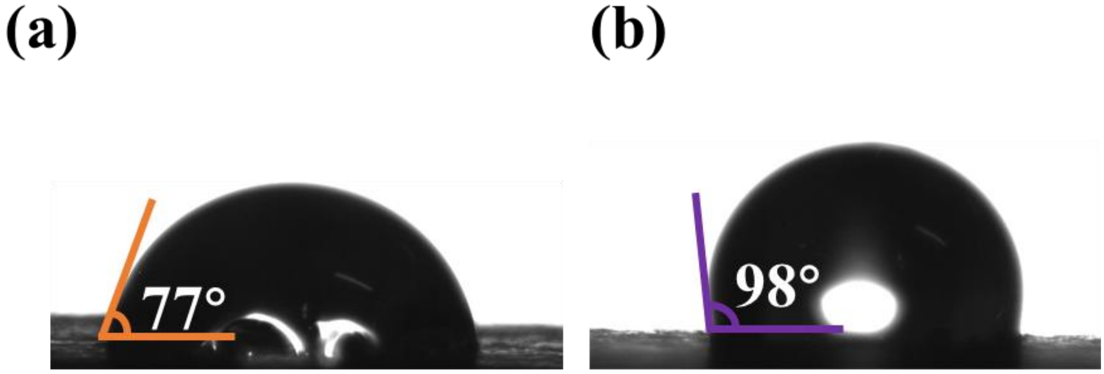

Figure S28. Contact angles of 0.1 M $KHCO_3$ aqueous electrolyte on the surface of (a) Cu/Cu_2O and (b) Cu/Cu_2O-Sb_2O_3-15.

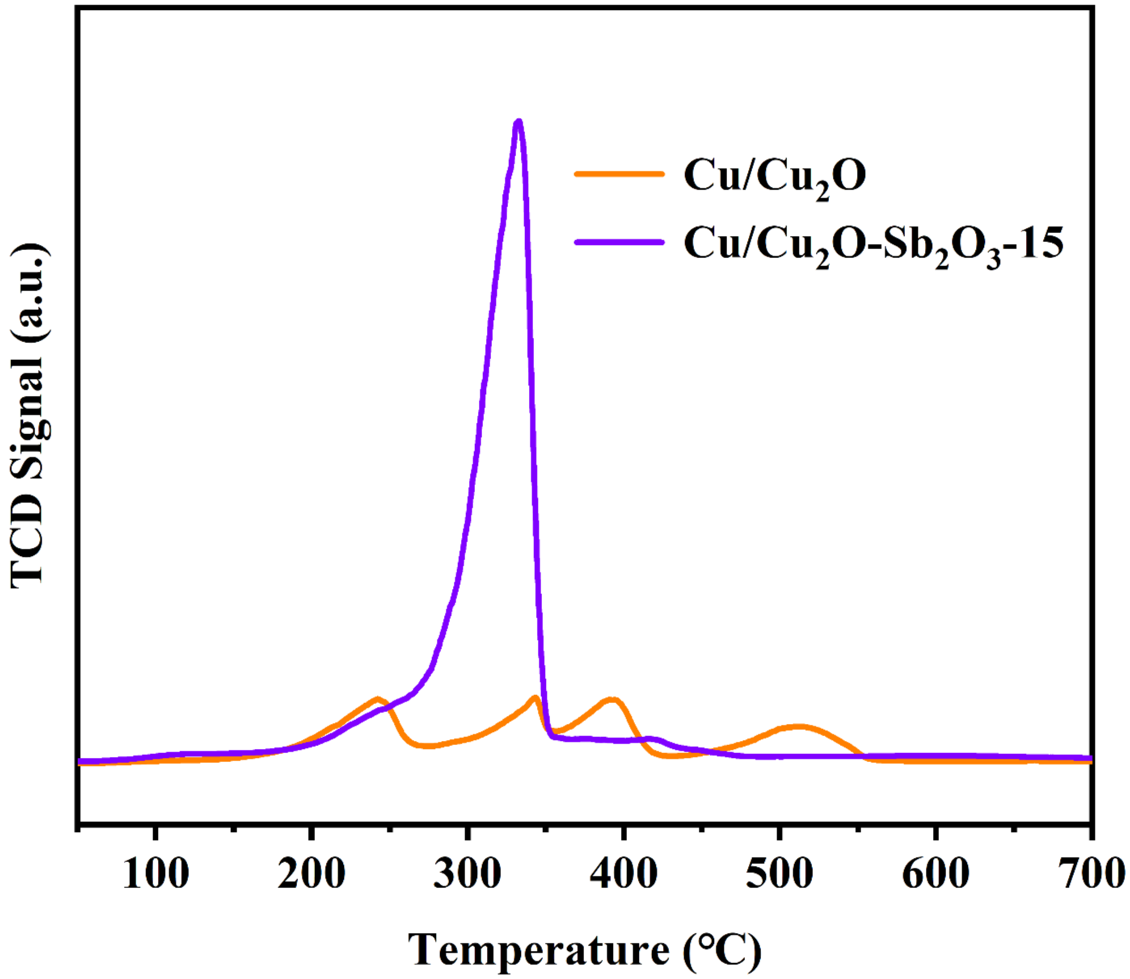

Figure S29. CO TPD spectra of Cu/Cu_2O and Cu/Cu_2O-Sb_2O_3-15 catalysts.

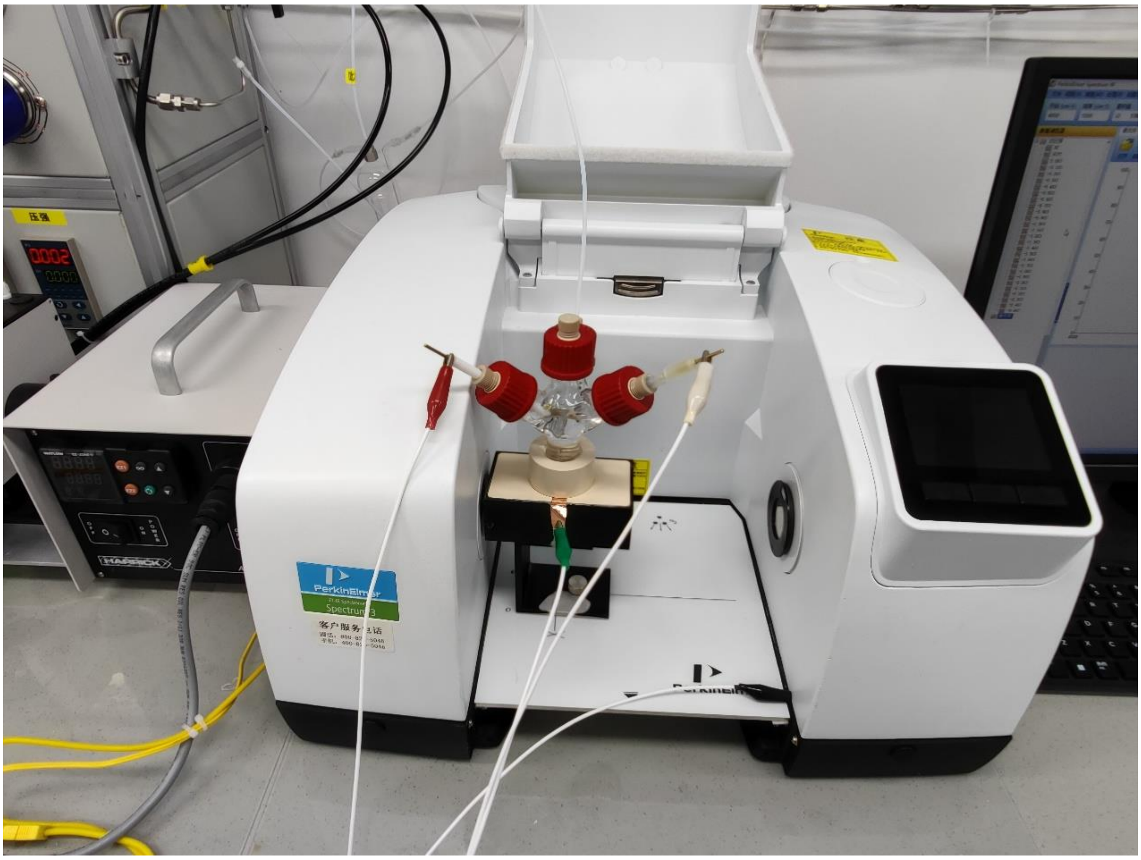

Figure S30. Digital photograph of in situ ATR-IR device.

Table S1. Comparison of $Cu/Cu_2O-Sb_2O_3$-15 as cathode catalyst for rechargeable aqueous $Zn-CO_2$ batteries.

Catalysts	OCP (V)	Electrolyte	Discharge products	P_{max} (mW cm^{-2})	Stability (h)	Refs
NiPG	-	0.1M $KHCO_3$	CO	0.6	13	[2]
Ir@Au	0.71	0.8M $KHCO_3$	CO	-	30	[3]
SAs-Ni-NC	-	0.5M $KHCO_3$	CO	1.4	32	[4]
Fe single-atom catalyst	0.73	0.8M $KHCO_3$	CO	0.6	25	[5]
CoPc@DNHCS-8	0.75	0.5M $NaHCO_3$	CO	1.02	40	[6]
Fe-SA/BNC	0.51	0.8M $KHCO_3$	CO	1.18	80	[7]
**$Cu/Cu_2O-Sb_2O_3$-15**	**0.731**	**1M $KHCO_3$**	**CO**	**3.01**	**140**	**This work**

Table S2. Actual molar ratios of Sb/Cu and actual metal loadings on the catalysts were measured by ICP-OES analysis.

**Catalysts**	**Cu wt.%**	**Sb wt.%**	**Sb/Cu molar ratio**
Cu/Cu_2O	91.23	0	0
Cu/Cu_2O-Sb_2O_3-05	84.99	4.25	4.492%
Cu/Cu_2O-Sb_2O_3-10	79.36	7.94	8.993%
**Cu/Cu_2O-Sb_2O_3-15**	**76.99**	**11.55**	**13.456%**
Cu/Cu_2O-Sb_2O_3-20	73.84	14.77	18.632%
Cu/Cu_2O-Sb_2O_3-25	65.93	16.48	22.876%
Cu/Cu_2O-Sb_2O_3-30	61.36	18.41	28.973%

Table S3. The amount of each phase in the three-phase heterojunction Cu/Cu_2O-Sb_2O_3-15 catalyst was calculated from XRD data.

Crystallographic plane	Peak Area	Proportion%	Summarizing%
Sb_2O_3(121)	17179	11	11 (Sb_2O_3)
Cu_2O(111)	74512	47	
Cu_2O(200)	11501	7	57 (Cu_2O)
Cu_2O(220)	4552	3	
Cu(111)	40773	26	32 (Cu)
Cu(200)	10043	6	

Table S4. Summary of the reported Cu-based electrocatalysts for CO_2 electro-reduction reaction to CO in recent years with H-cell.

Electrocatalysts	Potentials (V vs. RHE)	j_{co} (mA cm^{-2})	FE(%)	Ref.
**Cu/Cu_2O-Sb_2O_3-15**	**-1.3**	**-18.76**	**96.3**	**This work**
CuSAs/NC	-0.7	-5	92	[8]
Cu-N_4-NG	-1.0	-4	80.6	[9]
Cu_5Pd_5	-0.87	-7.8	88	[10]
Sn/Cu	-0.6	-5.43	82.7	[11]
Sb-Cu	-1.1	-6	82	[12]
Cu/In$(OH)_3$	-1.0	-10.1	89	[13]
C_3N_4/Cu_2O-FeO	-0.865	-4.65	84.4	[14]
Cu-HF	-1.5	-12.7	63.2	[15]
Cu-Au-HF	-1.5	-8.0	67.2	[15]
Cu-Ni-HF	-1.5	-13.6	77.5	[15]
CuO/$Cu_{4:3}$@BC	-0.7	-9.2	90.3	[16]
Cu@Cu_2O	-0.8	-11.1	87.6	[17]
Cu/Ag	-0.8	-1.50	89.1	[18]
Cu@SYS	-0.8	-13.2	78	[19]
Cu-CNT	-0.9	-3.5	80	[20]
CuFe/NC	-0.4	-2.1	95.5	[21]

Table S5. The formation energy of Sb-O doped Cu/Cu_2O with different doping sites.

	**Cu/Cu_2O-Sb_2O_3-1**	**Cu/Cu_2O-Sb_2O_3-2**	**Cu/Cu_2O-Sb_2O_3-3**
Total energy/eV	-473.941	-474.341	-474.512

Supplementary References

[1] W. Zheng, J. Yang, H. Chen, Y. Hou, Q. Wang, M. Gu, F. He, Y. Xia, Z. Xia, Z. Li, B. Yang, L. Lei, C. Yuan, Q. He, M. Qiu, X. Feng, *Advanced Functional Materials* **2020**, *30*, 1907658.

[2] R. Yang, J. Xie, Q. Liu, Y. Huang, J. Lv, M. A. Ghausi, X. Wang, Z. Peng, M. Wu, Y. Wang, *Journal of Materials Chemistry A* **2019**, *7*, 2575-2580.

[3] X. Y. Wang, J. F. Xie, M. A. Ghausi, J. Q. Lv, Y. Y. Huang, M. X. Wu, Y. B. Wang, J. N. Yao, *Adv Mater* **2019**, *31*, 0935-9648.

[4] W. Zheng, F. Chen, Q. Zeng, Z. Li, B. Yang, L. Lei, Q. Zhang, F. He, X. Wu, Y. Hou, *Nano-Micro Letters* **2020**, *12*, 134.

[5] T. Wang, X. Sang, W. Zheng, B. Yang, S. Yao, C. Lei, Z. Li, Q. He, J. Lu, L. Lei, L. Dai, Y. Hou, *Adv Mater* **2020**, *32*, 0935-9648.

[6] S. Gong, W. Wang, C. Zhang, M. Zhu, R. Lu, J. Ye, H. Yang, C. Wu, J. Liu, D. Rao, S. Shao, X. Lv, *Advanced Functional Materials* **2022**, *32*.

[7] S. Liu, M. Jin, J. Sun, Y. Qin, S. Gao, Y. Chen, S. Zhang, J. Luo, X. Liu, *Chemical Engineering Journal* **2022**, *437*, 135294.

[8] F. Yang, X. Mao, M. Ma, C. Jiang, P. Zhang, J. Wang, Q. Deng, Z. Zeng, S. Deng, *Carbon* **2020**, *168*, 528-535.

[9] C. Xu, X. Zhi, A. Vasileff, D. Wang, B. Jin, Y. Jiao, Y. Zheng, S.-Z. Qiao, *Small Structures* **2021**, *2*, 265-278.

[10] D. Chen, Y. Wang, D. Liu, H. Liu, C. Qian, H. He, J. Yang, *Carbon Energy* **2020**, *2*, 443-451.

[11] W. J. Dong, J. W. Lim, J. Y. Park, C. J. Yoo, S. Baek, W. S. Cho, W. Kim, J.-L. Lee, *Applied Surface Science* **2021**, *565*, 150460.

[12] H. Li, T.-W. Jiang, X. Qin, J. Chen, X.-Y. Ma, K. Jiang, X.-G. Zhang, W.-B. Cai, *ACS Catalysis* **2021**, *11*, 6846-6856.

[13] W. Li, Y. Yang, Z. Weng, S. Huo, *Journal of CO_2 Utilization* **2021**, *46*, 101470.

[14] G. W. Woyessa, J.-a. B. dela Cruz, M. Rameez, C.-H. Hung, *Applied Catalysis B-Environmental* **2021**, *291*, 120052.

[15] I. Merino-Garcia, J. Albo, P. Krzywda, G. Mul, A. Irabien, *Catalysis Today* **2020**, *346*, 34-39.

[16] Y. Zhou, X. Guo, X. Li, J. Fu, J. Liu, F. Hong, J. Qiao, *Journal of CO_2 Utilization* **2020**, *37*, 188-194.

[17] M. Wang, X. Ren, G. Yuan, X. Niu, Q. Xu, W. Gao, S. Zhu, Q. Wang, *Journal of CO_2 Utilization* **2020**, *37*, 204-212.

[18] W. J. Dong, C. J. Yoo, J. W. Lim, J. Y. Park, K. Kim, S. Kim, D. Lee, J.-L. Lee, *Nano Energy* **2020**, *78*, 105168.

[19] P. Huang, J. Chen, P. Deng, F. Yang, J. Pan, K. Qi, H. Liu, B. Y. Xia, *Journal of Catalysis* **2020**, *381*, 608-614.

[20] D. Karapinar, A. Zitolo, T. N. Huan, S. Zanna, D. Taverna, L. H. Galvao Tizei, D. Giaume, P. Marcus, V. Mougel, M. Fontecave, *Chemsuschem* **2020**, *13*, 173-179.

[21] F. Wang, H. Xie, T. Liu, Y. Wu, B. Chen, *Applied Energy* **2020**, *269*, 115029.
